# Development and validation of a machine learning model for predicting adverse prognosis in Wallerian degeneration patients based on clinical and imaging data

**DOI:** 10.3389/fneur.2026.1840010

**Published:** 2026-05-26

**Authors:** Zhiqi Yu, Xujie Wang, Ruiqi Tian, Siyue Chen, Wei Tang, Xuhui Liu

**Affiliations:** 1 Department of Neurology, Xinhua Hospital Affiliated with Dalian University, Shahekou, Dalian, Liaoning, China; 2 Department of Emergency ICU, The Affiliated Hospital of Qinghai University, Chengxi, Xining, Qinghai, China; 3 Department of Neurology, Yan'an University Medical College No.3 Affiliated Hospital, Weicheng, Xianyang, Shaanxi, China; 4 Department of Neurology, Fifth Affiliated Hospital of Xinjiang Medical University, Urumqi, Xinjiang, China; 5 Department of Neurology, The Second Hospital of Lanzhou University, Chengguan, Lanzhou, Gansu, China

**Keywords:** machine learning, poor prognosis risk, predictive model, SHAP, Wallerian degeneration

## Abstract

**Background:**

Wallerian degeneration (WD) is a neurodegenerative change that often leads to irreversible neurological dysfunction in the central nervous system, resulting in poor prognosis. Currently, there is a lack of predictive tools capable of estimating the risk of poor prognosis in WD patients. This study aims to develop a machine learning-based predictive model to assess the risk of poor prognosis in WD patients, with the outcome measure of modified Rankin Scale (mRS) assessed at least 3 months after WD diagnosis by imaging.

**Methods:**

The data for this study were sourced from Xinhua Hospital, affiliated with Dalian University. Clinical patient data were randomly split into a training set and a validation set in a 7/3 ratio. Based on the AIC/BIC criteria, the Boruta algorithm and multivariable logistic regression were used for variable selection, and eight machine learning models were constructed. The models were evaluated for discrimination, predictive accuracy, and clinical benefit using receiver operating characteristic (ROC) curves, calibration curves, and decision curve analysis (DCA). Additionally, SHAP values were used to systematically evaluate feature importance in the best-performing machine learning model.

**Results:**

A total of 285 WD patients were included in the study, with a median age of 63 years. Among them, 157 (55.09%) were male and 128 (44.91%) were female, and 161 (56.49%) patients had poor prognosis. Ten candidate predictors for poor prognosis in WD were identified through analysis: diabetes, hypertension, hyperlipidemia, atrial fibrillation, NIHSS score, medulla oblongata, periventricular region, centrum semiovale, subcortical volume 1,000, and the severity of WD. Seven of the ten predictors showed statistical significance, while three demonstrated a borderline association. Among the evaluated machine learning models, the AdaBoost model (AUC = 0.880) demonstrated the most stable performance in terms of discrimination, calibration, and clinical benefit. SHAP analysis indicated that NIHSS score, atrial fibrillation, and hypertension made significant contributions to predicting poor prognosis in WD patients.

**Conclusion:**

Our study successfully developed and validated a machine learning predictive model that integrates clinical indicators and imaging data to estimate the risk of poor prognosis in WD patients. This model may assist clinicians in identifying high-risk individuals and provide evidence-based guidance for early intervention and personalized management.

## Introduction

Wallerian degeneration (WD) refers to the process of anterograde degeneration that occurs when nerve fibers are cut or compressed, affecting the distal part (i.e., the axonal segment separated from the neuronal cell body) (1). Its prognosis typically manifests as motor dysfunction (e.g., paralysis) or sensory dysfunction (e.g., numbness or tingling). WD in the central nervous system (CNS) can lead to irreversible neurological dysfunction (2). Existing studies suggest that WD is one of the important causes of poor long-term neurological recovery in patients. It has been reported that the risk of poor functional prognosis (modified Rankin Scale scores of 4–6) significantly increases 3 months after the occurrence of WD (*P* = 0.046), indicating an overall poor functional prognosis (3). Moreover, active treatment measures can reduce the risk of poor WD prognosis, thereby improving the prognosis. A study on 20 patients with acute middle cerebral artery/internal carotid artery stroke found that early identification of imaging biomarkers (such as ADC changes) could improve early prognosis prediction and provide a basis for selecting appropriate rehabilitation treatments and neuroprotective trials for patients, effectively improving their prognosis (4). Therefore, accurate early prediction tools can help with personalized assessment of WD prognosis, enhance clinical monitoring of high-risk patients, and develop more effective early rehabilitation plans to improve patients' quality of life.

The pathophysiological mechanisms of WD are multifactorial, involving brain hypoxia, vascular dysfunction, coagulation abnormalities, and inflammatory cascade reactions (5). Previous studies have identified several risk factors, such as the type and extent of neural injury, age, and neural repair capacity. Additionally, comorbidities such as hypertension, diabetes, and atrial fibrillation, as well as imaging indicators (e.g., lesion location, regional ratio), are associated with the prognosis of WD (6). However, the predictive ability of traditional regression models remains limited. These models typically assume linear relationships, making it difficult to handle high-dimensional data and capture complex nonlinear interactions in WD prognosis.

Machine learning (ML) algorithms can effectively handle nonlinear relationships and extract hidden patterns from complex, high-dimensional data (7). In recent years, ML has made significant progress in predicting the prognosis of neurological diseases and critical care conditions, particularly in spontaneous intracerebral hemorrhage, ischemic stroke, and traumatic brain injury (8, 9). Algorithms such as AdaBoost, gradient boosting, and ensemble methods have demonstrated superior performance compared to traditional methods (10). However, research on WD prognosis prediction is still limited, and existing prediction methods have not been sufficiently validated.

WD prognosis assessment is an important clinical endpoint that generates personalized risk evaluations. However, existing methods rely on linear assumptions or limited feature integration and often lack interpretable risk assessments. Given the limitations of current evidence and the difficulty in capturing non-linear relationships between high-dimensional clinical, laboratory, and imaging predictors, we developed and validated a ML model for early prediction of poor WD prognosis. Compared to traditional regression models, ML is better suited for handling non-linear relationships and the complex interactions between imaging, inflammation, and clinical features. We compared eight commonly used ML algorithms and conducted a comprehensive evaluation, including discrimination, calibration, and decision analysis utility, by integrating SHAP explainability. Finally, we selected the best-performing algorithm, providing an interpretable, prognosis-driven tool that translates model outputs into clinically meaningful risk assessments and clinical decision support.

## Methods

**Figure 1:**
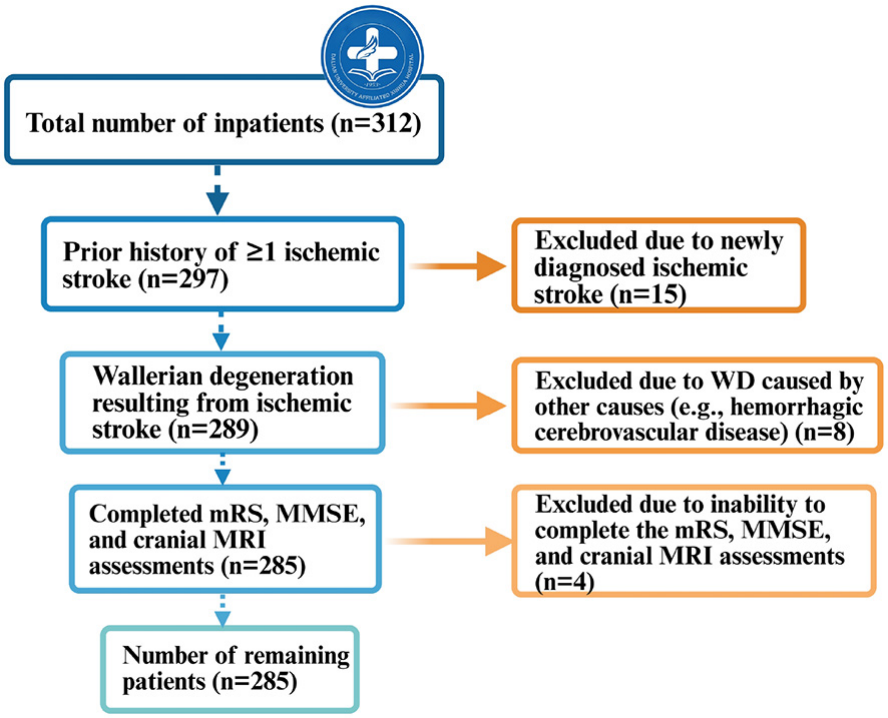
Flow diagram of the selection of patients with WD after IS.

This retrospective observational study was conducted at Xinhua Hospital, affiliated with Dalian University, following the STROBE (Strengthening the Reporting of Observational Studies in Epidemiology) guidelines, supplemented by the TRIPOD (Transparent Reporting of a multivariable prediction model for Individual Prognosis Or Diagnosis) checklist (Supplementary Table S1). Clinical and imaging data of all patients diagnosed with WD due to Ischemic stroke (IS) at the Department of Neurology, Xinhua Hospital, affiliated with Dalian University, from January 2022 to June 2024, were retrospectively collected. The data were extracted from the hospital's electronic medical record system. After strict inclusion and exclusion criteria, a total of 285 patients were included in the study. During the study period, 312 patients with WD, diagnosed through imaging or clinical assessment, were initially screened. Of these, 15 cases were excluded due to a diagnosis of newly onset IS, 8 were excluded due to other causes (e.g., hemorrhagic cerebrovascular diseases) leading to WD, and 4 were excluded due to the inability to cooperate with mRS, MMSE scoring, and cranial MRI. Ultimately, 285 patients were included in the analysis. The study protocol was reviewed and approved by the Ethics Committee of Xinhua Hospital, affiliated with Dalian University (Ethics No: 2024-12-01). Given that this is a retrospective analysis based on anonymized data, and the data do not include identifiable patient information, informed consent was waived. All study procedures adhered to institutional and national privacy and confidentiality standards. Figure 1 provides a flowchart summarizing the screening and exclusion process of the integrated study.

The study population included patients aged 18 or older who were hospitalized due to recurrent IS and diagnosed with WD through cranial computed tomography (CT) or magnetic resonance imaging (MRI). The inclusion criteria were: (1) age ≥18 years; (2) a history of ≥1 previous IS; (3) WD confirmed by cranial MRI. The exclusion criteria were: (1) new-onset IS; (2) WD caused by non-ischemic stroke; (3) inability to cooperate with the mRS, MMSE scales, or cranial MRI; After screening, a total of 285 patients were included and randomly allocated to the training cohort (199 patients) and the validation cohort (86 patients) in a 7:3 ratio for model development and internal validation. The split was performed using scikit-learn's train_test_split (X, y, test_size = 0.3, random_state = 42, stratify = y), executed in Python 3.9.7 with scikit-learn 1.0.2 and numpy 1.21.4. The age variable takes 26 unique integer values across 285 patients, reflecting the institutional de-identification protocol (whole-year integer truncation with quantization, consistent with the HIPAA Safe Harbor method). The age variable was not selected by Boruta (normHits = 0, mean importance = −0.327) and does not enter the final model.

WD was diagnosed based on predefined imaging criteria by two experienced neuroradiologists (each with >5 years of neuroimaging experience) who independently reviewed all cranial MRI scans. WD was identified as hyperintense signal along the expected course of degenerating white matter tracts on T2-weighted and/or FLAIR sequences, distal to the primary ischemic lesion. Disagreements were resolved by consensus. The readers were blinded to clinical outcome status at imaging interpretation.

The primary outcome was the occurrence of WD with poor prognosis, defined by the mRS score: mRS ≤ 2 was classified as a good prognosis, and mRS > 2 was classified as a poor prognosis. A total of 36 candidate predictors were collected, including demographic factors (age, gender, smoking, hypertension, diabetes, hyperlipidemia, atrial fibrillation) and scale assessments: (1) National Institutes of Health Stroke Scale (NIHSS) score on admission; (2) Mini-Mental State Examination (MMSE) and modified Rankin Scale (mRS) after imaging confirmation of WD; and imaging features (size of the ischemic stroke lesion, location and number of layers involved in WD, degree/severity of WD, width of the cerebral peduncle, and area ratio).

Operational definitions of key variables: 'Subcortical volume 100′ (SV 1000) was defined as a binary variable indicating whether the total subcortical structural volume (including basal ganglia, thalamus, and internal capsule) exceeded 1,000 mm3, as measured by automated volumetric segmentation using FreeSurfer (version 7.1; ICC = 0.92). 'Severity of WD' was classified as a binary variable based on T2/FLAIR signal: severe WD was defined as confluent hyperintensity extending across ≥2 contiguous anatomical levels (κ = 0.78). 'AIWD' (Asymmetry Index of Wallerian Degeneration) was calculated as the ratio of the difference to the sum of bilateral cerebral peduncle cross-sectional areas.

The outcome mRS was assessed at a minimum of three months after imaging confirmation of WD, representing the follow-up functional status. This is distinct from the baseline mRS recorded at the time of WD imaging confirmation, which was collected as a candidate predictor variable. The baseline predictor mRS (Table 1, ‘MRS' row: 148/285 = 51.93%) and the follow-up outcome mRS (161/285 = 56.49%) are measured at different time points and represent distinct clinical constructs. The temporal sequence is illustrated in Supplementary Figure S1.

**Table 1:** Baseline characteristics of the training and validation cohorts in patients with poor prognosis of WD.

	[ALL] *N* = 285	Test *N* = 86	Train *N* = 199	*p* overall
**Grouped**	0.56 (0.50)	0.55 (0.50)	0.57 (0.50)	0.683
Type stroke, *n* (%)				0.113
NHS	147 (51.58%)	51 (59.30%)	96 (48.24%)	
HS	138 (48.42%)	35 (40.70%)	103 (51.76%)	
Time WD, *n* (%)				1.000
≤ 2 months	135 (47.37%)	41 (47.67%)	94 (47.24%)	
>3 months	150 (52.63%)	45 (52.33%)	105 (52.76%)	
Sex, *n* (%)				0.418
Female	128 (44.91%)	35 (40.70%)	93 (46.73%)	
Male	157 (55.09%)	51 (59.30%)	106 (53.27%)	
**Age (year)**	63.00 [40.00; 99.00]	40.00 [40.00; 99.00]	85.00 [40.00; 99.00]	0.032
MRS > 2, *n* (%)				0.967
No	137 (48.07%)	42 (48.84%)	95 (47.74%)	
Yes	148 (51.93%)	44 (51.16%)	104 (52.26%)	
Hypertension, *n* (%)				0.417
No	138 (48.42%)	38 (44.19%)	100 (50.25%)	
Yes	147 (51.58%)	48 (55.81%)	99 (49.75%)	
Smoking, *n* (%)				1.000
No	148 (51.93%)	45 (52.33%)	103 (51.76%)	
Yes	137 (48.07%)	41 (47.67%)	96 (48.24%)	
Hyperlipidemia, *n* (%)				0.928
No	142 (49.82%)	42 (48.84%)	100 (50.25%)	
Yes	143 (50.18%)	44 (51.16%)	99 (49.75%)	
Diabetes mellitus, *n* (%)				0.091
No	149 (52.28%)	52 (60.47%)	97 (48.74%)	
Yes	136 (47.72%)	34 (39.53%)	102 (51.26%)	
Atrial fibrillation, *n* (%)				1.000
No	134 (47.02%)	40 (46.51%)	94 (47.24%)	
Yes	151 (52.98%)	46 (53.49%)	105 (52.76%)	
PV, *n* (%)				0.308
No	141 (49.47%)	47 (54.65%)	94 (47.24%)	
Yes	144 (50.53%)	39 (45.35%)	105 (52.76%)	
TL, *n* (%)				0.593
No	151 (52.98%)	43 (50.00%)	108 (54.27%)	
Yes	134 (47.02%)	43 (50.00%)	91 (45.73%)	
BG, *n* (%)				0.597
No	141 (49.47%)	40 (46.51%)	101 (50.75%)	
Yes	144 (50.53%)	46 (53.49%)	98 (49.25%)	
FL, *n* (%)				0.283
No	148 (51.93%)	40 (46.51%)	108 (54.27%)	
Yes	137 (48.07%)	46 (53.49%)	91 (45.73%)	
PL, *n* (%)				0.446
No	144 (50.53%)	40 (46.51%)	104 (52.26%)	
Yes	141 (49.47%)	46 (53.49%)	95 (47.74%)	
No	141 (49.47%)	37 (43.02%)	104 (52.26%)	
Yes	144 (50.53%)	49 (56.98%)	95 (47.74%)	
CSO, *n* (%)				0.113
No	147 (51.58%)	51 (59.30%)	96 (48.24%)	
Yes	138 (48.42%)	35 (40.70%)	103 (51.76%)	
SV 1,000, *n* (%)				0.712
No	156 (54.74%)	49 (56.98%)	107 (53.77%)	
Yes	129 (45.26%)	37 (43.02%)	92 (46.23%)	
SV 0, *n* (%)				0.886
No	146 (51.23%)	43 (50.00%)	103 (51.76%)	
Yes	139 (48.77%)	43 (50.00%)	96 (48.24%)	
MCA, *n* (%)				0.029
No	156 (54.74%)	56 (65.12%)	100 (50.25%)	
Yes	129 (45.26%)	30 (34.88%)	99 (49.75%)	
ACA, *n* (%)				0.058
No	142 (49.82%)	35 (40.70%)	107 (53.77%)	
Yes	143 (50.18%)	51 (59.30%)	92 (46.23%)	
PCA, *n* (%)				0.742
No	130 (45.61%)	41 (47.67%)	89 (44.72%)	
Yes	155 (54.39%)	45 (52.33%)	110 (55.28%)	
PC, *n* (%)				0.844
No	135 (47.37%)	42 (48.84%)	93 (46.73%)	
Yes	150 (52.63%)	44 (51.16%)	106 (53.27%)	
LLP, *n* (%)				0.828
No	148 (51.93%)	46 (53.49%)	102 (51.26%)	
Yes	137 (48.07%)	40 (46.51%)	97 (48.74%)	
UPP, *n* (%)				0.563
No	135 (47.37%)	38 (44.19%)	97 (48.74%)	
Yes	150 (52.63%)	48 (55.81%)	102 (51.26%)	
MO, *n* (%)				0.039
No	131 (45.96%)	48 (55.81%)	83 (41.71%)	
Yes	154 (54.04%)	38 (44.19%)	116 (58.29%)	
Sev WD, *n* (%)				0.274
No	150 (52.63%)	50 (58.14%)	100 (50.25%)	
Yes	135 (47.37%)	36 (41.86%)	99 (49.75%)	
**SVS (%)**	13,379.46 [824.96; 38,393.11]	10,578.42 [2,420.71; 37,452.73]	16,895.77 [204.76; 38,900.62]	0.969
**Lev WD**	3.00 [1.00; 15.00]	7.00 [1.00; 15.00]	2.00 [1.00; 15.00]	0.913
**Deg WD**	0.48 [0.05; 0.66]	0.46 [0.05; 0.66]	0.51 [0.05; 0.66]	0.968
**MMSE**	20.00 [17.00; 29.00]	21.00 [18.00; 29.00]	20.00 [17.00; 29.00]	0.485
**AIWD**	0.14 [0.03; 0.43]	0.24 [0.03; 0.43]	0.13 [0.03; 0.42]	0.912
**NIHSS**	8.00 [2.00; 23.00]	8.00 [3.00; 24.00]	8.00 [2.00; 22.50]	0.407

Type Stroke, Type of Stroke; HS, Hemorrhagic Stroke; NHS, Non-hemorrhagic Stroke; Time WD, Time since diagnosis of WD; MRS > 2, Modified Rankin Scale > 2; PV, Periventricular; TL, Temporal Lobe; BG, Basal Ganglia; FL, Frontal Lobe; PL, Parietal Lobe; OL, Occipital Lobe; CSO, Centrum Semiovale; SV 1,000/SV 0, Subcortical Volume [1,000/0]; MCA, Middle Cerebral Artery; ACA, Anterior Cerebral Artery; PCA, Posterior Cerebral Artery; PC, Posterior Circulation; LLP, Left Limb Paresis; UPP, Upper Limb Paresis; MO, Medulla Oblongata; Sev WD, Severity of WD; SVS, Signs and Symptoms; Lev WD, Level of WD; Deg WD, Degree of WD; AIWD, Asymmetry Index of Wallerian Degeneration.

First, the Boruta algorithm was used to select relevant variables, with the most correlated variables being retained for further analysis. The rejected features were excluded from further analysis. The Boruta algorithm was applied exclusively to the training cohort (*n* = 199) to prevent information leakage from the validation set. A backward stepwise multivariate logistic regression was then applied to select important predictive variables and assess their impact on the target outcome. As a result, ten predictors with non-zero coefficients were identified, all of which were retained based on AIC/BIC optimization for poor prognosis prediction in WD. To check for multicollinearity, the variance inflation factor (VIF) was also assessed, and no significant multicollinearity was found.

Missing data handling: Among the 36 candidate variables, 28 core variables (including all 10 final predictors) had complete data for all 285 patients. Eight non-core independent variables exhibited missing values at rates of 6% or lower (range: 0.7–5.6%). None of these were selected by Boruta. The final model was based on complete data without imputation (Supplementary Table S2).

Variable retention in the backward stepwise procedure was guided by Akaike Information Criterion (AIC) and Bayesian Information Criterion (BIC) rather than individual *p*-values. The full 10-variable model had the lowest AIC (187.9); removing any variable increased the AIC (Supplementary Table S3). Seven variables achieved conventional significance (*p* < 0.05), while three (NIHSS *p* = 0.077, HTN *p* = 0.062, HLD *p* = 0.060) showed borderline associations but were retained because their inclusion minimized AIC.

The ten independent predictors (diabetes, hypertension, hyperlipidemia, atrial fibrillation, NIHSS score, medulla oblongata, periventricular, centrum semiovale, subcortical volume 1,000, and the severity of WD) were used to construct eight machine learning models: RandomForest, XGBoost, LightGBM, GradientBoosting, AdaBoost, LogisticReg LinearReg, Lasso, and GaussianNB. The performance of each model was comprehensively assessed through discrimination, calibration, and clinical utility. Discrimination was quantified using the area under the receiver operating characteristic curve (AUC), precision-recall analysis, and confusion matrix-based metrics (including sensitivity, specificity, accuracy, recall, F1 score, positive predictive value (PPV), and negative predictive value (NPV)). Calibration was evaluated using calibration plots, Hosmer-Lemeshow goodness-of-fit tests, and Brier scores. The validation-cohort AUC was pre-specified as the primary metric for final model selection, supplemented by cross-validation stability, calibration performance, and net clinical benefit via decision curve analysis as secondary selection criteria.

Five-fold stratified cross-validation was performed on the training cohort to obtain unbiased estimates of model discrimination. Bootstrap resampling (*n* = 1,000) was performed on the validation cohort to derive 95% confidence intervals for all performance metrics. Learning curves were generated to assess overfitting. To evaluate incremental predictive value, AdaBoost was compared against a baseline NIHSS-only logistic regression model using the DeLong test. The complete analytical pipeline is illustrated in Supplementary Figure S1.

Among these eight models, AdaBoost demonstrated the strongest robustness and generalizability, consistently exhibiting high accuracy, sensitivity, and specificity in both cohorts. To improve interpretability, the Shapley additive explanation (SHAP) method was used to explain AdaBoost, quantifying the contribution of each predictor to the model's output. The SHAP summary plot revealed that NIHSS score, atrial fibrillation, and hypertension made the most significant contributions, followed by lesion locations in the medulla oblongata, periventricular region, centrum semiovale, hyperlipidemia, severity of WD, diabetes, and subcortical volume 1,000, highlighting the multifactorial pathophysiology of WD prognosis.

Categorical variables are presented as counts and percentages, while continuous variables are summarized as medians with interquartile ranges. Statistical comparisons between groups were performed using chi-square (χ^2^) or Fisher's exact test for categorical variables, and Mann–Whitney U test for continuous variables. Logistic regression models reported odds ratios (OR) and their 95% confidence intervals (CIs). All statistical analyses were performed using R software (version 4.2.1) and Python (version 3.9.7), with a two-tailed *p*-value < 0.05 considered statistically significant.

## Results

A total of 285 WD patients were included in this study, with 157 male patients (55.09%) and 128 female patients (44.91%). The median age was 63.00 years (range: 40.00–99.00). The overall incidence of poor prognosis in WD patients was 56.49% (*n* = 161). Patients were divided into the training cohort (199 patients) and the validation cohort (86 patients) in a 7:3 ratio. As shown in Table 1, the training and validation cohorts were generally comparable in most baseline characteristics. However, statistically significant differences were observed for age (*p* = 0.032), MCA territory involvement (*p* = 0.029), and medulla oblongata involvement (*p* = 0.039). These imbalances are acknowledged as a limitation. However, there were significant differences between the poor prognosis group and the good prognosis group, as shown in Supplementary Table S4. The proportion of patients with hypertension (61.49% vs. 38.51%, *p* < 0.001), hyperlipidemia (59.63% vs. 40.37%, *p* < 0.001), and atrial fibrillation (66.46% vs. 33.54%, *p* < 0.001) was higher in the poor prognosis group. Higher NIHSS scores were more common in the poor prognosis group (*p* < 0.001). In terms of imaging features, the incidence of poor prognosis was higher when WD occurred in the medulla oblongata (70.81% vs. 29.19%, *p* < 0.001), while lesions in the centrum semiovale (36.65% vs. 63.35%) and periventricular region (37.27% vs. 62.73%) were significantly negatively correlated with poor prognosis. The severity of WD also showed a significant difference between the two groups, with more severe cases in the poor prognosis group (61.49% vs. 38.51%, *p* < 0.001). Additionally, subcortical volume 1000 was significantly negatively correlated with poor prognosis (32.30% vs. 67.70%, *p* < 0.001). Overall, these findings suggest that clinical and imaging features are associated with poor prognosis in WD and may serve as valuable candidate indicators for the predictive model.

### Ranking of features importance

**Figure 2:**
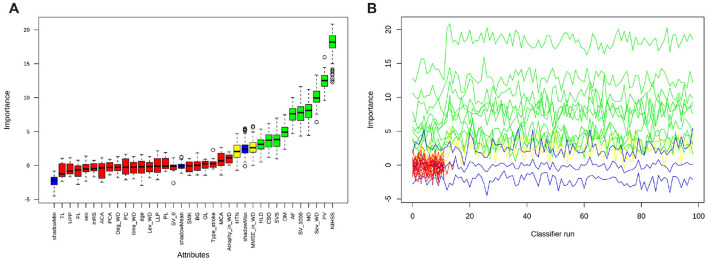
Variables importance screening in Boruta. **(A)** Ranking of importance scores for each predictor variable. **(B)** Reference threshold line for determining the importance of predictor variables.

To identify the variables most related to the prognosis of WD, we used the Boruta algorithm to screen 36 variables. After 500 iterations and Bonferroni correction, 10 variables (HLD, CSO, HTN, DM, AF, MO, Sev-WD, PV, and NIHSS) with the most significant predictive ability were selected to build our model (Figure 2). Figure 2A shows the box plot of feature importance scores, which displays the distribution of the importance of different features. According to the box plot, the green features, such as HLD, CSO, HTN, Sev-WD, and NIHSS, are located at higher scores, indicating that these features are the most important for predicting WD prognosis. Figure 2B shows the change curves of feature importance over 500 classifier runs. Green features, such as HLD, CSO, and HTN, exhibit small fluctuations in multiple iterations, indicating that these features have stable importance across different classifier runs. In contrast, other colors (such as red and blue) represent features with larger fluctuations, suggesting that their predictive abilities are less stable across different runs.The average importance scores and feature selection decisions are shown in Supplementary Table S5.

### Multivariate logistic regression to identify independent predictors of poor prognosis in WD

**Figure 3:**
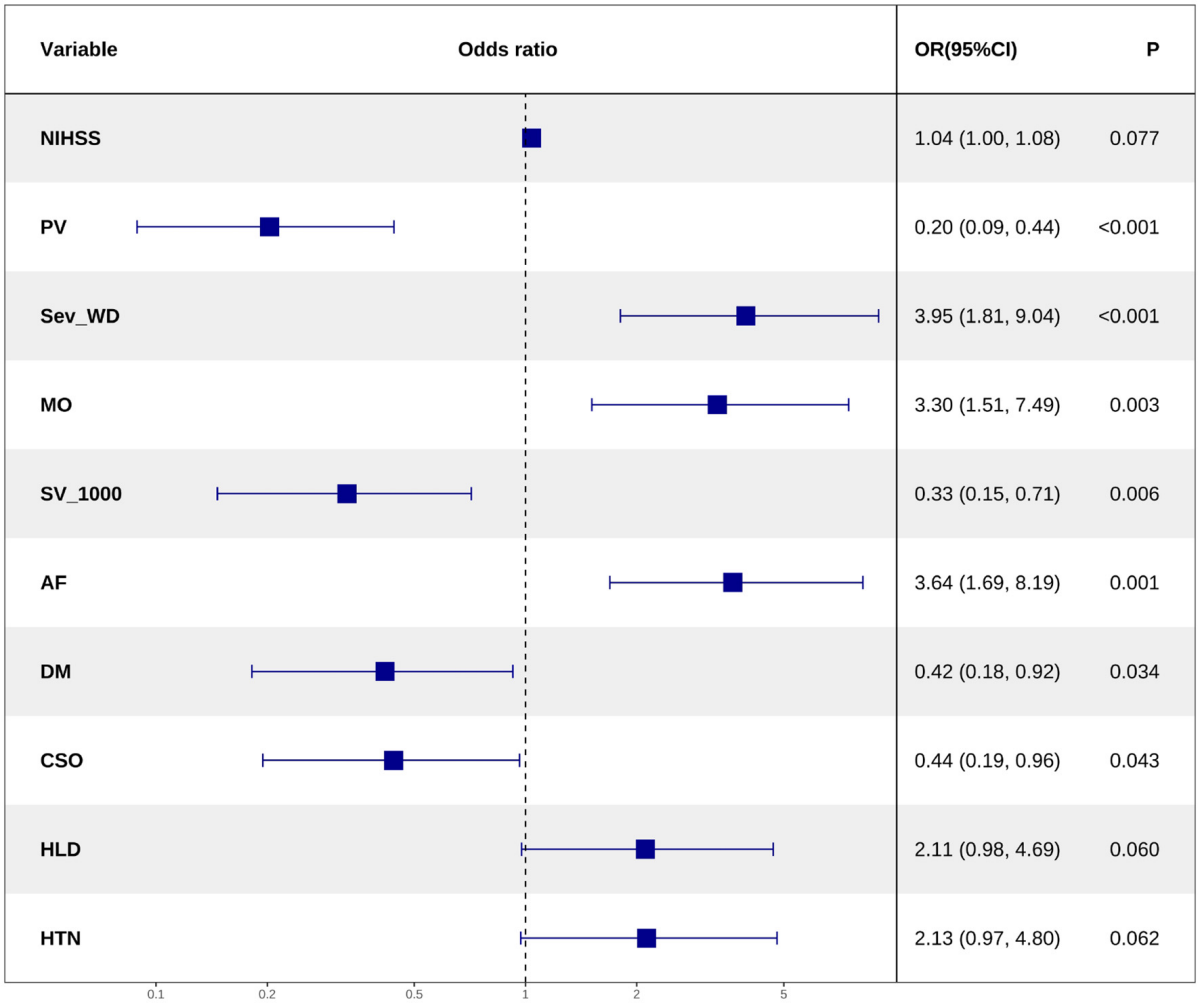
Forest plot of multivariate logistic regression analysis for poor prognosis of WD. The forest plot shows the odds ratios (ORs) and 95% confidence intervals (CIs) for each predictor in the multivariate model.

To further identify independent predictors of poor prognosis in WD, all the variables selected by the Boruta algorithm were input into a backward stepwise multivariate logistic regression model. As shown in Figure 3, 10 variables were retained in the final model based on AIC optimization. NIHSS score showed a positive association with poor prognosis (borderline significance) (OR = 1.039, 95% CI: 0.996–1.083, *p* = 0.077). Patients with hyperlipidemia (OR = 2.112, 95% CI: 0.968–4.610, *p* = 0.06), diabetes (OR = 0.417, 95% CI: 0.186–0.934, *p* < 0.05), hypertension (OR = 2.127, 95% CI: 0.962–4.704, *p* = 0.062), and atrial fibrillation (OR = 3.640, 95% CI: 1.661–7.978, *p* < 0.01) had a higher likelihood of poor prognosis. Infarction sites in the periventricular region (PV) (OR = 0.203, 95% CI: 0.092–0.451, *p* < 0.001), medulla oblongata (MO) (OR = 3.305, 95% CI: 1.492–7.319, *p* < 0.01), and centrum semiovale (CSO) (OR = 0.439, 95% CI: 0.198–0.973, *p* < 0.05) were strong risk factors, and the severity of WD was also a significant risk factor (OR = 3.948, 95% CI: 1.775–8.784, *p* < 0.001). In contrast, increased subcortical volume was a protective factor for poor prognosis. For every 1,000 mm3 increase in subcortical volume, the risk of poor prognosis decreased to 0.328 times the original (OR = 0.328, 95% CI: 0.150–0.721, *p* < 0.01). These results support a multifactorial etiology, including comorbidities, clinical, and imaging features. Multicollinearity was assessed using the variance inflation factor (VIF), and all variables had VIF values below 2 and close to 1, indicating that there is little linear dependence between these variables. Further details are provided in Supplementary Table S6.

### Machine-learning model development and evaluation

**Figure 4:**
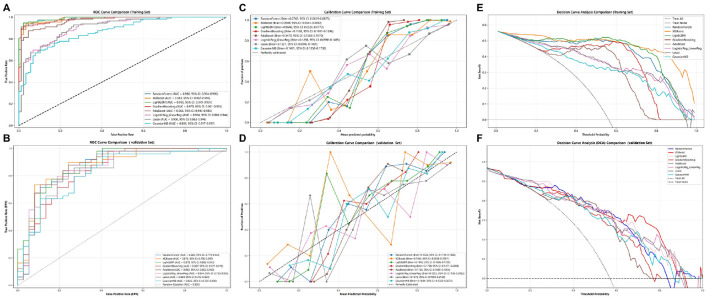
Performance of eight ML models for predicting the prognosis of WD. **(A)** ROC curves in the training cohort. **(B)** ROC curves in the validation cohort. **(C)** Calibration plot in the training cohort. **(D)** Calibration plot in the validation cohort. **(E)** Decision-curve analysis (DCA) in the training cohort. **(F)** DCA in the validation cohort.

Based on the 10 features selected through the two variable selection methods mentioned above, we constructed 8 ML models, including RandomForest, XGBoost, LightGBM, GradientBoosting, AdaBoost, Logistic Regression, Lasso, and GaussianNB, to predict poor prognosis in WD patients. To improve the model's performance and generalizability, hyperparameter optimization was performed, with detailed results provided in Supplementary Table S7. The optimized hyperparameters for each ML model are listed in Table 2. The performance of these 8 ML models in the training and validation sets, including ROC curves, calibration curves, and DCA, is shown in Figure 4. In the training cohort, AdaBoost (AUC = 0.966, 95% CI: 0.943–0.985) exhibited high discrimination. In the validation cohort, AdaBoost (AUC = 0.880, 95% CI: 0.802–0.950) demonstrated the strongest generalizability, while XGBoost, LightGBM, and RandomForest showed moderate performance (Figures 4A, B; Table 3). In addition to AUC, calibration analysis confirmed good consistency between the predicted probabilities and actual observed probabilities (Figures 4C, D), and decision curve analysis indicated that AdaBoost consistently provided the greatest net clinical benefit across a wide range of threshold probabilities (Figures 4E, F). Further evaluation metrics, including precision-recall (PR) parameters, Brier scores (Supplementary Tables S8, S9), and confusion matrix-based metrics (Supplementary Figure S2), such as sensitivity, specificity, predictive values, accuracy, recall, and F1 score (Supplementary Table S10; Supplementary Figures S3, S4), further validated the superior robustness of AdaBoost compared to other algorithms. A direct comparison of AUC distributions across models (Supplementary Table S11; Supplementary Figure S5) further supports these findings. Overall, these results suggest that AdaBoost is the most balanced model with good generalizability, supported by cross-validation with multiple evaluation metrics.

**Table 2:** Performance metrics of the ML models in the training and validation cohorts.

Model	Dfclass	Sensitivity	Specificity	Pos Pred Value	Neg Pred Value	Precision	Recall	F1
RandomForest	dev	0.955	0.931	0.947	0.942	0.947	0.955	0.951
XGBoos	dev	0.938	0.943	0.955	0.921	0.955	0.938	0.946
LightGBM	dev	0.955	0.954	0.964	0.943	0.964	0.955	0.960
GradientBoosting	dev	0.955	0.851	0.892	0.937	0.892	0.955	0.922
AdaBoost	dev	0.902	0.874	0.902	0.874	0.902	0.902	0.902
LogisticReg LinearReg	dev	0.830	0.793	0.838	0.784	0.838	0.830	0.834
Lasso	dev	0.839	0.793	0.839	0.793	0.839	0.839	0.839
GaussianNB	dev	0.804	0.713	0.783	0.738	0.783	0.804	0.793
RandomForest	vad	0.776	0.784	0.826	0.725	0.826	0.776	0.800
XGBoos	vad	0.776	0.784	0.826	0.725	0.826	0.776	0.800
LightGBM	vad	0.796	0.784	0.83	0.744	0.830	0.796	0.812
GradientBoosting	vad	0.837	0.649	0.759	0.750	0.759	0.837	0.796
AdaBoost	vad	0.858	0.757	0.824	0.800	0.824	0.857	0.840
LogisticReg LinearReg	vad	0.837	0.73	0.804	0.771	0.804	0.837	0.820
Lasso	vad	0.816	0.757	0.816	0.757	0.816	0.816	0.816
GaussianNB	vad	0.816	0.676	0.769	0.735	0.769	0.816	0.792

XGBoost, eXtreme Gradient Boosting; LightGBM, Light Gradient Boosting Machine; GradientBoosting, Gradient Boosting Machine; AdaBoost, Adaptive Boosting; Lasso, Least Absolute Shrinkage and Selection Operator; GaussianNB, Gaussian Naive Bayes.

**Table 3:** Pairwise DeLong test *p*-values for AUC comparisons among eight machine learning models in the training and validation cohorts.

	dfclass	RF	XGB	LGBM	GBT	ADA	LR	Lasso	GNB
RF	dev	1	0.285757	0.209873	0.067058	0.001383	0.000006	0.00001	0
XGB	dev	0.285757	1	0.085568	0.290676	0.001502	0.000008	0.000012	0
LGBM	dev	0.209873	0.085568	1	0.015826	0.001497	0.000013	0.000019	0
GBT	dev	0.067058	0.290676	0.015826	1	0.017314	0.000072	0.000112	0.000001
ADA	dev	0.001383	0.001502	0.001497	0.017314	1	0.00008	0.000157	0.000001
LR	dev	0.000006	0.000008	0.000013	0.000072	0.00008	1	0.296883	0.010161
Lasso	dev	0.00001	0.000012	0.000019	0.000112	0.000157	0.296883	1	0.012655
GNB	dev	0	0	0	0.000001	0.000001	0.010161	0.012655	1
RF	vad	1	0.592878	0.595599	0.288076	0.426314	0.388046	0.327674	0.17624
XGB	vad	0.592878	1	0.898589	0.106924	0.719704	0.255274	0.202254	0.15137
LGBM	vad	0.595599	0.898589	1	0.008406	0.889317	0.360111	0.308194	0.187636
GBT	vad	0.288076	0.106924	0.008406	1	0.04219	0.855417	0.938969	0.803335
ADA	vad	0.426314	0.719704	0.889317	0.04219	1	0.199862	0.164467	0.090157
LR	vad	0.388046	0.255274	0.360111	0.855417	0.199862	1	0.303317	0.365646
Lasso	vad	0.327674	0.202254	0.308194	0.938969	0.164467	0.303317	1	0.524282
GNB	vad	0.17624	0.15137	0.187636	0.803335	0.090157	0.365646	0.524282	1

Random Forest, RF; XGBoos, XGB; Light GBM, LGBM; Gradient Boosting, GBT; AdaBoost, ADA; LogisticReg LinearReg, LR; Gaussian NB, GNB.

**Figure 5:**
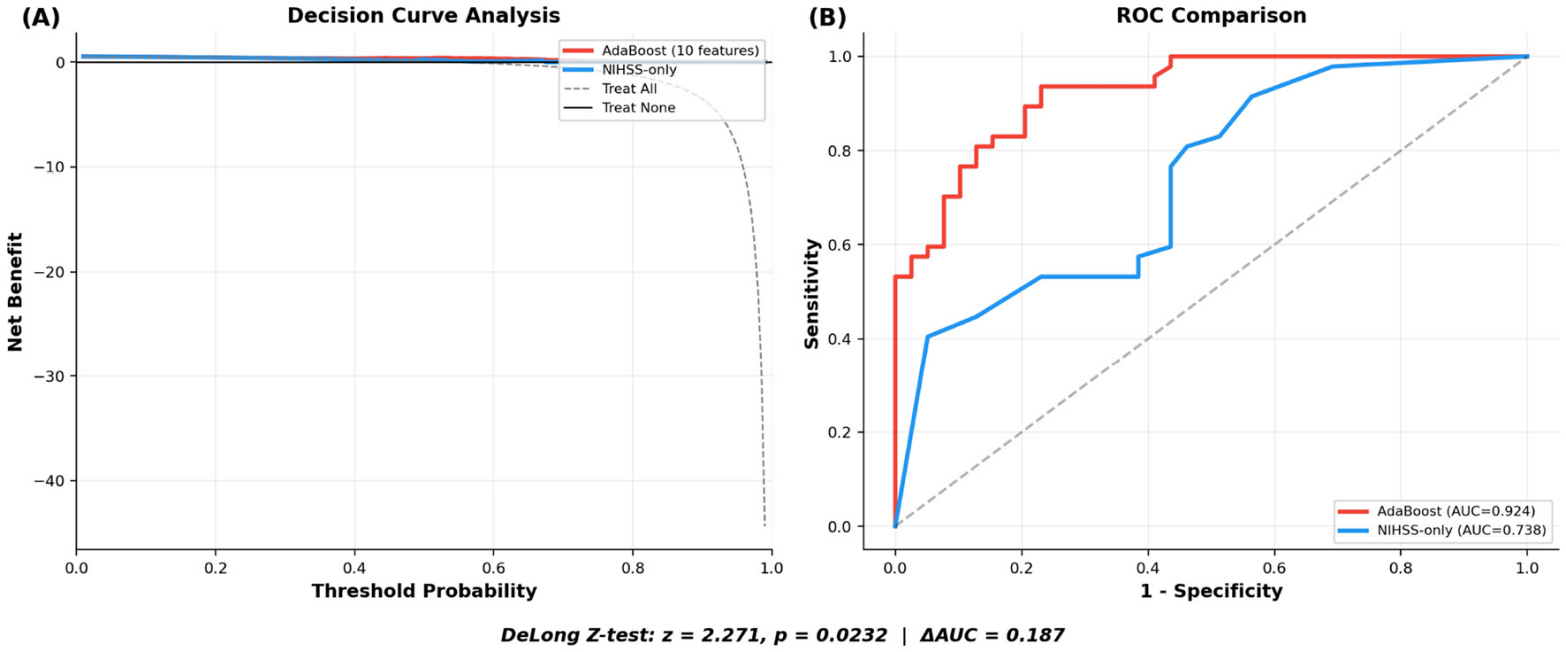
Comparison of AdaBoost (10 features) versus NIHSS-only logistic regression model in the validation cohort. **(A)** Decision curve analysis (DCA) comparing net clinical benefit. AdaBoost (red) provides greater net benefit than NIHSS-only (blue) across threshold probabilities 0.2–0.8. Gray dashed line = treat all; black line = treat none. **(B)** ROC curve comparison. AdaBoost AUC = 0.924 vs. NIHSS-only AUC = 0.738; DeLong Z-test: z = 2.271, *p* = 0.023, confirming statistically significant superiority.

**Figure 6:**
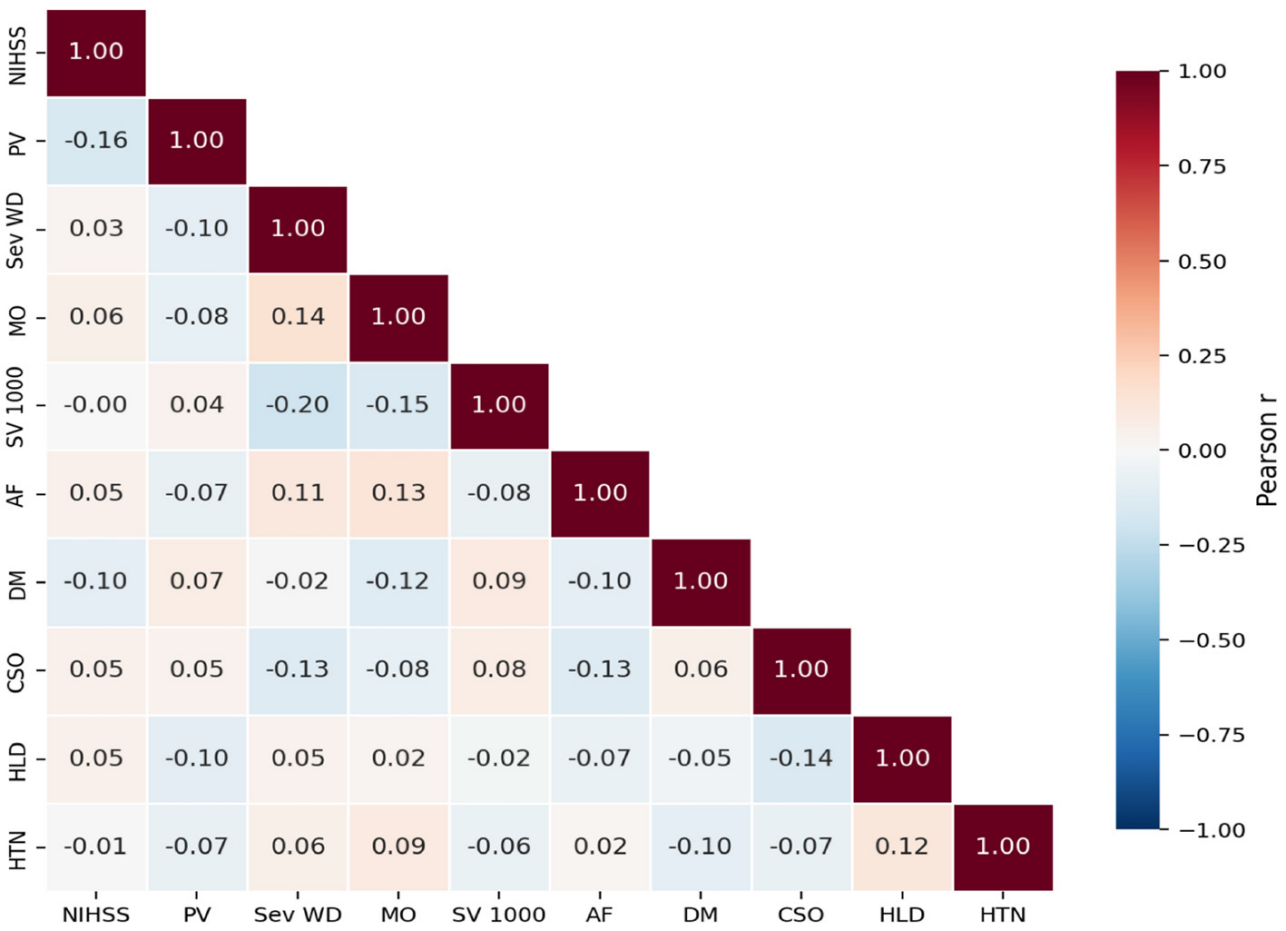
Correlation matrix of 10 selected predictors. Lower-triangle Pearson correlation heatmap. All pairwise correlations are weak to moderate (|r| < 0.35), consistent with variance inflation factor (VIF) values below 2.0 (Supplementary Table S6). This confirms minimal multicollinearity among the selected predictors and supports the validity of SHAP-based interpretations.

Five-fold cross-validation on the training cohort confirmed AdaBoost's stable discriminative performance (mean CV-AUC = 0.930, SD = 0.046; Supplementary Table S12; Supplementary Figure S6). Learning curve analysis demonstrated a moderate training-validation AUC gap (0.086) within acceptable range for ensemble methods (Supplementary Figure S7). Bootstrap-derived 95% CIs for all metrics are reported in Supplementary Table S15 and Supplementary Figure S8. Furthermore, AdaBoost (AUC = 0.924) significantly outperformed a NIHSS-only model (AUC = 0.738; DeLong z = 2.271, p = 0.023; Table 4; Figures 5, 6). The Youden-Index-optimized threshold for AdaBoost was 0.531 (J = 0.661), yielding sensitivity = 0.796 and specificity = 0.865, compared with the default 0.5 threshold (sensitivity = 0.837, specificity = 0.784; Supplementary Table S14, Supplementary Figure S9). An inverse-probability-of-split-weighted sensitivity analysis using age, MCA territory, and medulla oblongata as weighting covariates confirmed robustness: the propensity score C-statistic was 0.548, and the IPW-weighted validation AUC was 0.897 (95% bootstrap CI: 0.815–0.958), virtually identical to the unweighted AUC of 0.897 (95% CI: 0.825–0.957; Supplementary Tables S15, S16; Supplementary Figure S10). Comparison with a NIHSS + age + hypertension composite logistic regression (AUC = 0.578) confirmed the substantial superiority of AdaBoost (ΔAUC = 0.318; Supplementary Figure S11). Pairwise DeLong tests with Bonferroni correction for 15 comparisons (α = 0.0033) revealed no significant differences among top-performing models (all adjusted *p* > 0.05; Supplementary Table S17; Supplementary Figure S12), consistent with limited power at n = 86 (minimum detectable ΔAUC = 0.140; Supplementary Table S18). The Hosmer–Lemeshow test showed acceptable calibration for parametric models (LogisticReg: *p* = 0.767; GaussianNB: *p* = 0.395) but significant statistics for AdaBoost (*p* = 0.001; Supplementary Table S19, Supplementary Figure S13), consistent with known behavior of boosting algorithms (11).

**Table 4:** Comparison of AdaBoost (10 features) versus NIHSS-only logistic regression. DeLong Z-test: z = 2.271, *p* = 0.023.

Model	AUC	Sensitivity	Specificity	F1	DeLong-z	DeLong-p
NIHSS-only (LR)	0.7379	0.9149	—	0.7679	2.271	0.0232
AdaBoost (10 features)	0.9244	0.9362	—	0.8381	—	—

Additional validation analyses are provided in the Supplementary materials. The distribution of all 10 selected predictors stratified by outcome group is shown in Supplementary Figure S14, confirming clear separation for AF, MO, PV, CSO, Sev WD, SV 1000, and NIHSS, consistent with their high Boruta importance scores. Supplementary Figure S15 presents calibration curves for all models in the validation cohort, with AdaBoost demonstrating acceptable calibration along the diagonal. Supplementary Figure S16 shows precision-recall curves with average precision (AP) scores; AdaBoost achieved AP = 0.881. Supplementary Table S20 reports complete confusion matrix-derived metrics (TP, TN, FP, FN, sensitivity, specificity, PPV, NPV, accuracy, F1, and AUC) for all models in both cohorts. Supplementary Table S21 presents optimal decision thresholds determined by Youden Index maximization for all models in the validation cohort; AdaBoost achieved an optimal threshold of 0.525 with Youden J = 0.705 (Supplementary Figure S17). Supplementary Table S22 presents subgroup analyses of AdaBoost model performance stratified by sex, hypertension, atrial fibrillation, diabetes, medulla oblongata involvement, and WD severity, demonstrating consistent model performance across clinical subgroups (all subgroup AUCs ≥ 0.848). DeLong pairwise AUC comparisons between all models (Supplementary Figure S18) confirmed that most top-performing models did not differ significantly (all *p* > 0.05), consistent with the limited statistical power of n = 86. The age distribution analysis (Supplementary Figure S19) illustrates the limited granularity of the age variable (26 unique values) and the significant cohort imbalance (training median = 85 vs. validation median = 40, *p* = 0.032). Supplementary Figure S20 provides a visual summary of missing data, confirming that all 10 core predictors and 15 other non-core variables had 100% data completeness, while 8 non-core variables exhibited missing rates ≤ 6%. Supplementary Figure S21 presents AdaBoost native feature importance (Gini importance), with NIHSS showing the highest importance score (0.628), consistent with its top ranking in SHAP analysis.

### AdaBoost as the optimal predictive model and SHAP-based interpretation

**Figure 7:**
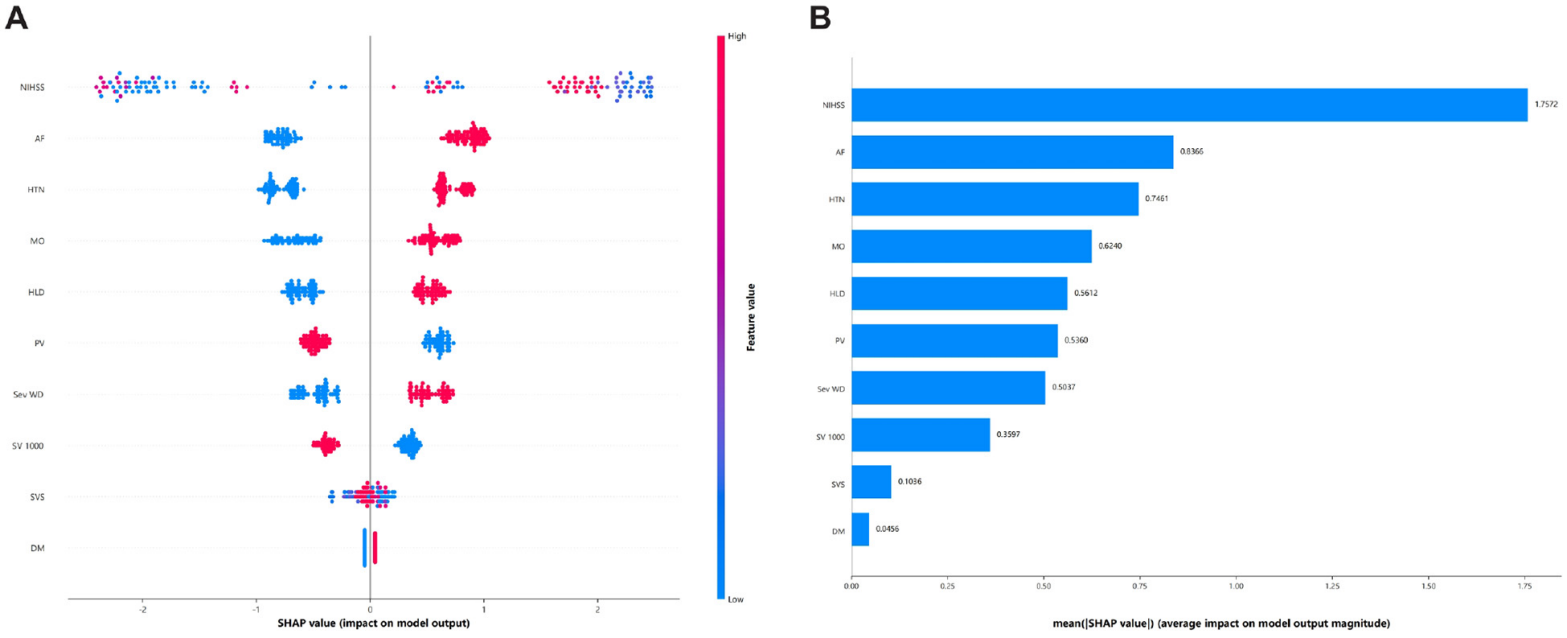
The figure illustrates the impact of each feature on the model's prediction results using SHAP. **(A)** shows the distribution of the feature impacts, and **(B)** presents the importance ranking of the features.

**Figure 8:**
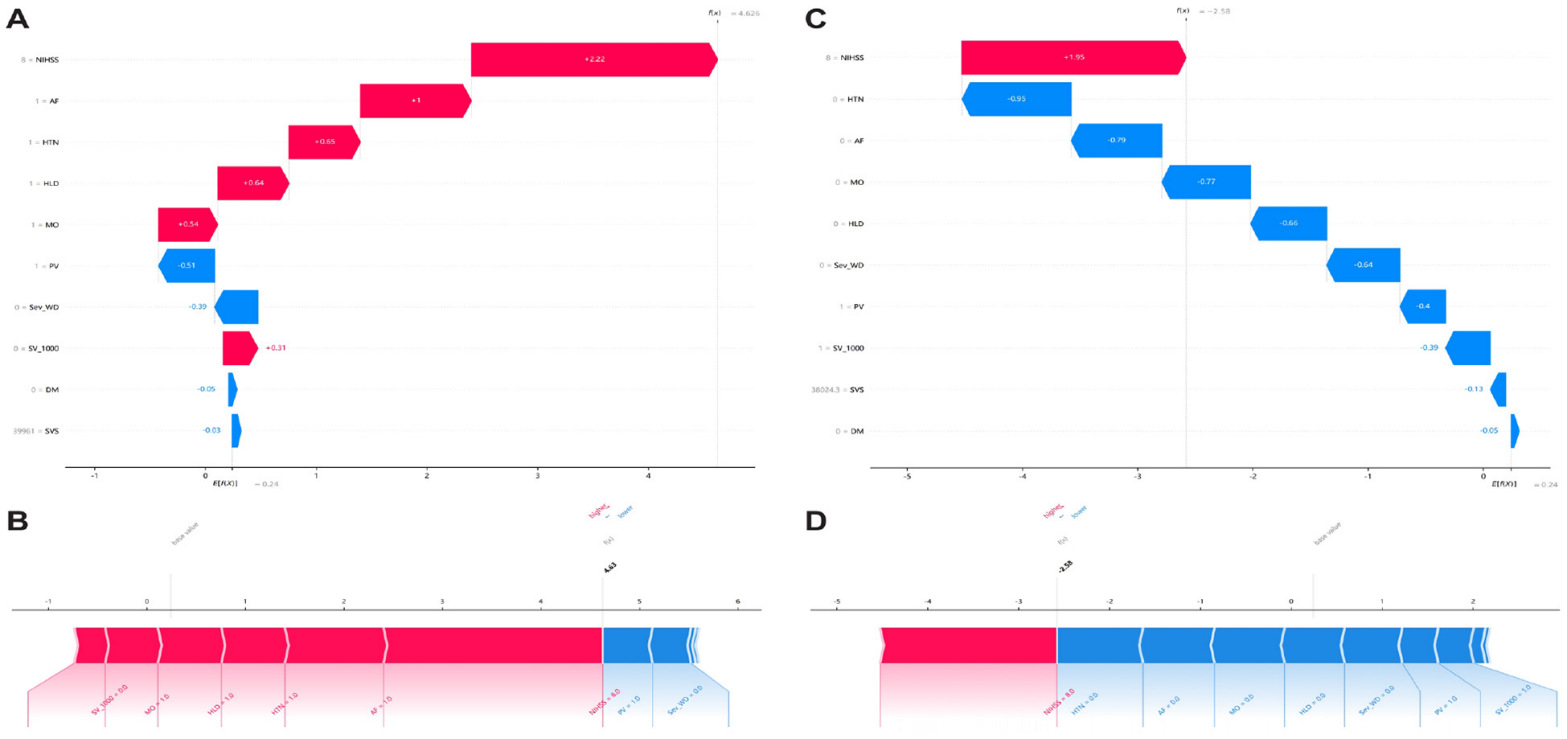
SHAP-based feature contribution analysis plot. **(A)** Contribution values of each feature to the model output in positive outcomes; **(B)** Distribution of feature contributions at the sample level in positive outcomes (waterfall plot). **(C)** Contribution values of each feature to the model output in negative outcomes; **(D)** Distribution of feature contributions at the sample level in negative outcomes (waterfall plot).

To further explore the features that predict poor prognosis in WD, we built an AdaBoost model and used SHAP and waterfall plots to visualize the distribution of variable contributions, measuring the importance of each variable through ranking gains. In the AdaBoost model (Figure 7), Figure 7A shows the SHAP value distribution for each feature, where positive values indicate a positive impact on the prediction result, and negative values indicate a negative impact. Different colors of the points represent different values of the features, with red indicating higher feature values and blue indicating lower feature values. The order of the features reflects their contribution to the model prediction, and we observed that features such as NIHSS, AF, and HTN have higher importance, indicating their significant impact on predicting WD patient prognosis. Figure 7B shows the average SHAP value for each feature, representing the average impact of each feature on the model output. From the figure, it is clear that NIHSS is the most important feature, with an average SHAP value of 1.7572, significantly higher than other features. Additionally, in positive outcomes, NIHSS = 8 received a positive weight vector of 2.22, AF = 1 received a positive weight of 1, and HTN = 1 received a positive weight of 0.65 (Figures 8A, B). In negative outcomes, HTN = 0 received a negative weight of −0.95, AF=0 received a negative weight of −0.79, and MO=0 received a negative weight of −0.77 (Figures 8C, D). This further suggests that NIHSS score, hypertension, and atrial fibrillation are important potential factors influencing poor prognosis in WD. To further enhance clinical interpretability, we also provided SHAP dependency plots for key predictive variables (Supplementary Figure S22), which visually identify the value ranges for each feature that significantly contribute to the WD prognosis risk and show how this impact changes across different feature values. Moreover, we created radar and SHAP summary plots to show the importance of different features in the model and the SHAP values of each feature on the model output, finding that NIHSS, AF, and HTN showed significant impact in the SHAP summary plot on the right side (Supplementary Figure S23A). Multi-sample decision plots (Supplementary Figure S23B) show the relationships and interaction effects between different features. From purple to red, the influence of features on model predictions is illustrated, showing that the interaction effects between NIHSS and features like AF, HTN, etc., may have a significant impact on prediction results. Additionally, the SHAP heatmap (Supplementary Figure S23C) and interaction effect network plot (Supplementary Figure S23D) illustrate the contribution of the selected predictive factors to each individual in the optimal AdaBoost model and the interaction strength and influence network between features. These visualizations show how each feature influences the prediction results of different individuals, enhancing clinical interpretability. After considering the individual effects of these factors on the model output, we also studied how they interact to jointly affect the model's predictions (Supplementary Figure S24; Supplementary Table S23). The table shows the five strongest feature interactions: the interaction between HTN and MO (strength: 0.103), HLD and Sev WD (strength: 0.082), Sev WD and NIHSS (strength: 0.0687), AF and NIHSS (strength: 0.0675), and AF and MO (strength: 0.0499). The strength of these interactions indicates that the combination of AF and NIHSS with these features has a significant effect on the model's predictions. In conclusion, the AdaBoost model not only demonstrates reliable and robust predictive performance with good generalizability but also maintains high interpretability through SHAP analysis, providing a clinically valuable tool for prognostic assessment in WD patients.

## Discussion

So far, ML models for predicting the prognosis of WD patients are still relatively limited. In this study, we proposed and validated an interpretable ML model to address this important clinical challenge. We used the Boruta algorithm to select ten key variables from a broad range of clinical features to construct multiple ML models. Among the tested algorithms, the AdaBoost model achieved the strongest balance between discrimination and calibration. Our key findings are summarized as follows: (1) The AdaBoost algorithm demonstrated the best predictive performance in the validation cohort (AUC = 0.88); (2) Shapley additive explanation (SHAP) analysis identified NIHSS score, atrial fibrillation, and hypertension as the most important predictors, followed by lesion locations in the medulla oblongata and periventricular region, hyperlipidemia, WD severity, diabetes, and subcortical volume 1,000 as the most influential predictors. Unlike previous studies on stroke that focused on the extent of traumatic neurodegenerative progression, our strict definition of poor prognosis outcomes and the inclusion of multimodal predictive factors may partially explain the differences in reported performance metrics and the optimality of different algorithms across various studies (12). The kernel-based framework of AdaBoost excelled particularly well in capturing non-linear interactions between risk factors, thus overcoming the limitations of traditional regression models. Notably, the probability-based performance evaluation confirmed the stability of the model, further enhancing its translational value in real-world clinical settings.

Our model identified the NIHSS score as one of the strong predictors of poor prognosis in WD. This is consistent with previous evidence, as higher scores generally indicate more severe neurological impairment and are associated with higher mortality and disability risks (13). For example, Puig et al. in a study combining clinical scale scores and acute ischemic lesions in specific areas of the corticospinal tract validated their value in predicting motor function recovery (14). Additionally, NIHSS scores often interact with other comorbidities in stroke patients, such as hypertension and diabetes, further impacting the neurorepair process and the progression of WD (15). These cumulative vulnerabilities may explain the consistent association between NIHSS scores and WD prognosis.

Hypertension is a common comorbidity in WD patients, accelerating endothelial damage and arteriosclerosis, which affects cerebral blood flow and oxygen supply, increasing the severity of neurological damage after a stroke. Impaired blood flow and persistent hypertension can exacerbate the progression of WD, reduce neurorepair capacity, and lead to poor prognosis (16). Hyperlipidemia, particularly elevated low-density lipoprotein (LDL) levels, is another key factor influencing WD prognosis. Hyperlipidemia promotes atherosclerosis, increases the risk of vascular occlusion and cerebral ischemia. Restricted blood flow exacerbates neuronal hypoxia, and hyperlipidemia may also trigger inflammatory responses, further accelerating WD progression (17). Hyperglycemia can directly damage nerve cells, inhibit neurorepair mechanisms, and promote neurodegeneration. Terada et al. demonstrated through immunohistochemistry and western blot analysis that diabetes causes delayed axonal degeneration and impairs the neurorepair process (18). Diabetes is also closely associated with arteriosclerosis, impaired blood flow, and insufficient oxygen supply, all of which may worsen WD (19). Furthermore, patients with diabetes have impaired immune function, increased infection risks, and find it more difficult to recover from WD, leading to a worse prognosis. Atrial fibrillation (AF) increases the risk of thromboembolic events due to abnormal heart rhythms, often resulting in reduced cerebral blood flow and triggering IS (20). Additionally, since AF is often treated with anticoagulation therapy, patients are at higher risk for brain hemorrhage, which complicates the relationship between WD and AF (21). WD patients with hypertension and AF show more pronounced abnormalities in cerebral hemodynamics and endothelial function, suggesting that there may be a synergistic effect between the two in the poor prognosis of WD (22).

Imaging features such as infarction sites (medulla oblongata, periventricular, centrum semiovale) and the severity of WD are also key predictors of high risk. In imaging diagnosis, infarctions located in the medulla oblongata carry the highest risk of progression, which may be due to the irreversible neuronal damage caused by WD following medullary infarction, especially affecting neural circuits controlling basic physiological functions. Since the medulla oblongata involves crucial functions such as breathing, heartbeat, and swallowing, its damage often indicates a poor prognosis, with patients at risk for long-term rehabilitation issues or even death. A previous case involving a 71-year-old male patient showed neurodegenerative changes in the medulla oblongata following a unilateral pons infarction, which was associated with poor prognosis (23, 24). In our cohort, infarctions located in the periventricular region, particularly in the periventricular white matter, may adversely affect the neurological prognosis. The periventricular white matter region contains many important neural pathways, especially the corticospinal tract (CST), so damage to this region and the occurrence of WD usually result in poor prognosis (24).The centrum semiovale is an important subcortical region of the brain, primarily composed of white matter fibers, and serves as a key conduit for transmitting information between different regions of the cerebral cortex. In WD, due to the degenerative changes in the brain's white matter, damage to the centrum semiovale often indicates more severe neuronal damage. If this area is compromised, the coordination of neural transmission and brain function will be affected, leading to more pronounced motor, cognitive, and other functional impairments, thereby impacting the prognosis. Studies have shown that in patients with minor IS, high-load white matter hyperintensities, particularly deep white matter lesions such as those in the centrum semiovale, are significantly associated with the degree of initial neurological impairment after stroke (25). Additionally, the severity of WD refers to the depth and extent of degenerative changes in nerve fibers, including demyelination, axonal rupture, and other processes. The severity of WD can be assessed through imaging techniques such as MRI (26). For instance, high signals (high signal on T2/FLAIR imaging) may indicate more severe neurodegeneration, typically involving major damage to axons and myelin. The severity of WD determines the degree of neurological impairment, with more severe WD often indicating prolonged functional loss and poorer rehabilitation prospects.

Decreased subcortical volume is often closely related to the severity of WD, and in our cohort, subcortical volume 1,000 was negatively correlated with poor prognosis in WD. After a stroke or other neurological damage, WD not only affects the area of injury but also spreads distally along neural pathways. In a study of epilepsy patients who underwent temporal lobectomy, volume reduction in subcortical structures such as the thalamus, putamen, and globus pallidus was observed, which was thought to be related to WD (27). As white matter undergoes degenerative changes, subcortical volume may gradually decrease, especially in regions such as the basal ganglia, thalamus, and internal capsule, leading to dysfunction in neural pathways and often indicating severe cognitive and motor impairments (28). Therefore, monitoring changes in subcortical volume through imaging can help predict the patient's neurological recovery and rehabilitation potential.

We agree that core ML components (model family, internal validation strategy, and performance metrics) are now widely applied in IS literature, and clinical translation of tools such as SHAP has been reported. For example, Yimin et al. used ML to analyze retinal images, providing a non-invasive, rapid, and efficient tool to assess the risk of ischemic and hemorrhagic strokes. The study used SHAP-based explanation methods, offering interpretability for ML model predictions, overcoming the limitations of traditional “black-box” models, and helping to increase trust and applicability in healthcare (29). Logistic regression still holds competitive value in certain datasets, especially in cases with relatively linear structure and fewer feature interactions [e.g., Bang et al. (30)]. Similarly, SVM performs strongly in some IS outcome prediction tasks [e.g., Wang et al. (7)]. Differences between studies often arise from outcome definitions, time windows, population severity, case mix, feature availability (especially imaging variables), sample size, class imbalance, preprocessing/imputation choices, and variations in hyperparameter tuning/validation protocols. In this context, our study aimed to compare multiple established algorithms using a unified feature set and evaluation framework, selecting the model with the most robust clinical applicability for predicting poor WD prognosis. In summary, these predictive factors highlight the multifactorial nature of WD prognosis, driven by interactions between clinical, neurological, physiological, imaging features, and laboratory biomarkers. The intersection of vascular dysfunction, systemic inflammation, coagulation imbalance, and metabolic disturbances provides a coherent mechanistic framework explaining the clinical heterogeneity of WD and the predictive validity of our model. Established composite prognostic scores for acute stroke (iScore, ASTRAL, DRAGON) were not included as comparators because these instruments were developed and validated exclusively for ischemic or hemorrhagic stroke populations and have not been validated for WD-specific prognosis. WD represents a secondary degenerative process with distinct pathophysiological mechanisms; direct application of these scores would constitute inappropriate extrapolation. Instead, AdaBoost was compared against NIHSS-only logistic regression and a NIHSS + age + hypertension composite, both substantially outperformed (ΔAUC ≥ 0.318).

Several methodological considerations merit discussion. Regarding SHAP interpretability: the TreeExplainer used for AdaBoost computes Shapley values under a feature independence assumption that is partially violated when predictors are correlated. Our interaction analysis (Supplementary Table S20) identified meaningful correlations (e.g., HTN–MO, AF–NIHSS). Individual SHAP values should therefore be interpreted with caution; the interaction analysis provides complementary context. Regarding model selection: AdaBoost was not the top-performing model in the training cohort, but was selected based on validation performance, confirmed by 5-fold cross-validation (mean AUC = 0.930). The training-validation AUC gap (0.086) indicates moderate overfitting, which learning curve analysis suggests would decrease with additional data. An unexpected finding was the apparently protective association between diabetes mellitus and favorable WD prognosis (crude OR = 0.477, adjusted OR = 0.637, 95% CI: 0.323–1.256). This paradox likely reflects confounding by treatment intensity: diabetic patients exhibited lower mean NIHSS scores (11.1 vs. 13.0) and lower prevalence of co-morbid atrial fibrillation (47.8% vs. 57.7%) and hypertension (46.3% vs. 56.4%), suggesting closer medical surveillance. Survivorship bias may also contribute. Future prospective studies should incorporate treatment intensity metrics to formally test this hypothesis. Regarding calibration, the Hosmer–Lemeshow test confirmed excellent fit for parametric models but significant statistics for AdaBoost, reflecting the known tendency of boosting algorithms to produce overly confident probability estimates; *post-hoc* calibration methods such as Platt scaling may be applied in clinical deployment. The choice between the default 0.5 threshold and the Youden-optimized 0.531 threshold should be guided by clinical context: the former favors sensitivity, the latter specificity (Supplementary Figure S25).

The main contribution of this study lies not in identifying entirely new risk factors but in quantifying and integrating multidimensional risks to address an early clinical endpoint—poor prognosis in WD—that has been under-researched. Specifically, (1) we used ML techniques to systematically integrate heterogeneous data, including imaging, coagulation, inflammation, and metabolic biomarkers, capturing non-linear contributions and joint patterns that traditional linear models often fail to reveal. (2) Methodologically, we performed rigorous feature selection and conducted a systematic comparison of eight ML algorithms, identifying AdaBoost as the optimal and most robust model. (3) In addition to assessing discrimination, we comprehensively evaluated the clinical utility of the model through calibration and decision curve analysis (DCA), and integrated SHAP interpretability to link model predictions with biologically reasonable patterns, ensuring transparency and clinical relevance. While experienced clinicians can identify these risk factors, the subjective integration of these factors into consistent, individualized probability estimates often involves bias. Therefore, our AdaBoost model is not intended to replace clinical judgment but serves as an objective, reproducible decision support tool, providing a transparent framework to help achieve early, targeted diagnosis and intervention by visualizing the synergistic effects of predictive factors.

However, there are several limitations to this study: (1) Since the study was conducted in a single center in China, the external generalizability of the results needs to be further validated in different racial and healthcare environments; (2) Despite statistical adjustments, potential confounding factors are difficult to completely avoid in observational studies; (3) Although the model performed well in internal validation, it still needs direct comparison with physician assessments before clinical application and should undergo large-scale, multicenter prospective studies.

Additional limitations include: (4) Clinical utility has not been demonstrated through comparison with physician predictions; a prospective blinded clinician accuracy study is needed before clinical deployment. (5) The validation cohort (*n* = 86) provides moderate statistical power; the 95% CI width of 0.148 for AUC reflects substantial uncertainty. (6) Class imbalance (56.49% positive) was not explicitly addressed through resampling; future work should explore threshold optimization via Youden Index or cost-sensitive approaches. (7) Age distribution showed limited granularity and significant imbalance between cohorts (training median 85 vs. validation median 40, *p* = 0.032), though age was not selected as a predictor. (8) The reported values [40; 99] represent the range (min–max), not the interquartile range. The TRIPOD checklist is provided in Supplementary Table S1. A further methodological limitation is the use of a single hold-out validation rather than fully nested cross-validation. (31) (BMC Bioinformatics, 2006) showed that nested cross-validation provides less biased generalization estimates; Vabalas et al. (32) (PLoS ONE, 2019) demonstrated that optimistic bias increases with smaller samples. Nested CV was not implemented because: (1) *n* = 285 limits feasible outer folds; (2) Boruta was applied exclusively within the training set; and (3) 5-fold CV partially mitigates overfitting. *Post-hoc* sample size assessment following Riley et al. (33) (BMJ, 2019) revealed that with 86 validation cases and SE (AUC) = 0.050, the minimum detectable ΔAUC at 80% power is 0.140; detecting ΔAUC = 0.05 would require approximately 292 patients, underscoring the need for external validation in a larger, multi-center cohort.

Future research is expected to further strengthen clinical translation. While SHAP enables sample-level explanations, this study mainly focused on model development and retrospective validation. Future studies will incorporate individual-level longitudinal trajectories (e.g., dynamic changes in imaging and laboratory parameters over time) to investigate how changes in patient status influence WD prognosis. We also plan to conduct prospective, multicenter validation and implementation studies to assess the real-world clinical impact of model-assisted decision-making. If externally and prospectively validated, this model could serve as a prognostic risk calculation tool to support WD prognosis assessment and guide personalized diagnosis, rehabilitation, and treatment, particularly by prioritizing early imaging and closer monitoring for high-risk patients.

## Conclusion

In this study, we developed and validated an interpretable and high-performing ML model—AdaBoost—for predicting the prognosis of WD patients. The model demonstrated excellent discrimination and calibration, and its strong clinical interpretability was achieved through SHAP analysis. By integrating key clinical and imaging features, the model provides a promising tool, pending external validation and prospective clinical evaluation, for prognostic risk assessment. Its translational potential lies in optimizing personalized management strategies and guiding timely interventions to improve the prognosis of this vulnerable population.

## Data Availability

The raw data supporting the conclusions of this article will be made available by the authors, without undue reservation.
