# Supplementary material for: Development and validation of a machine learning model for predicting adverse prognosis in Wallerian degeneration patients based on clinical and imaging data
Source: Front Neurol. 2026 May 26;17:1840010. doi: 10.3389/fneur.2026.1840010 (PMC13246421; doi:10.3389/fneur.2026.1840010)
Supplement: Supplementary file 1 [file Supplementary_file_1.pdf]

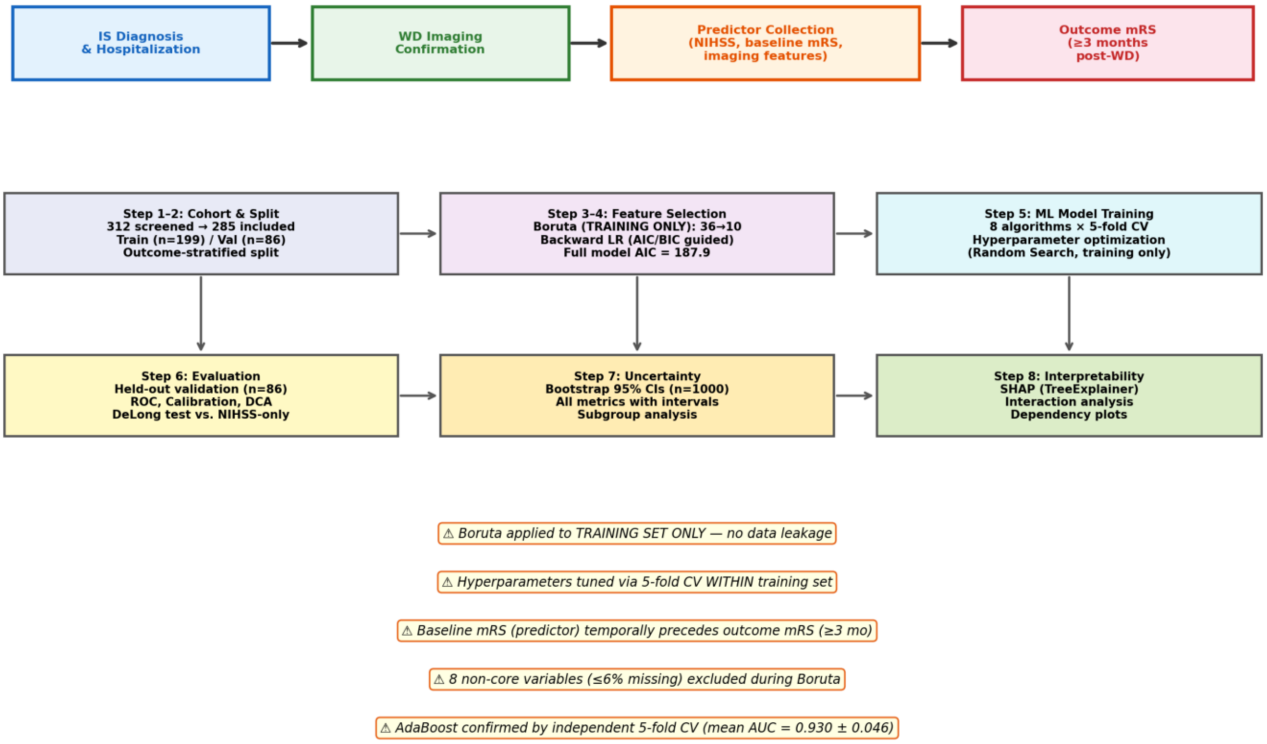

Figure S1 End-to-end analytical pipeline for machine learning model development and validation.
The diagram illustrates the temporal sequence of data collection (top) and the complete analytical pipeline (bottom). Key methodological safeguards are annotated: Boruta feature selection was applied exclusively to the training set (n = 199); hyperparameter tuning used 5-fold cross-validation within the training set; the validation cohort (n = 86) was reserved for final evaluation; and the predictor mRS (baseline) was measured before the outcome mRS (⩾3 months post-WD confirmation).The outcome (poor prognosis) was defined as an mRS score >2 during a follow-up period of at least 3 months (⩾3 months) after radiological confirmation of WD, thereby ensuring a temporal interval between the measurement of baseline predictors and outcome assessment.

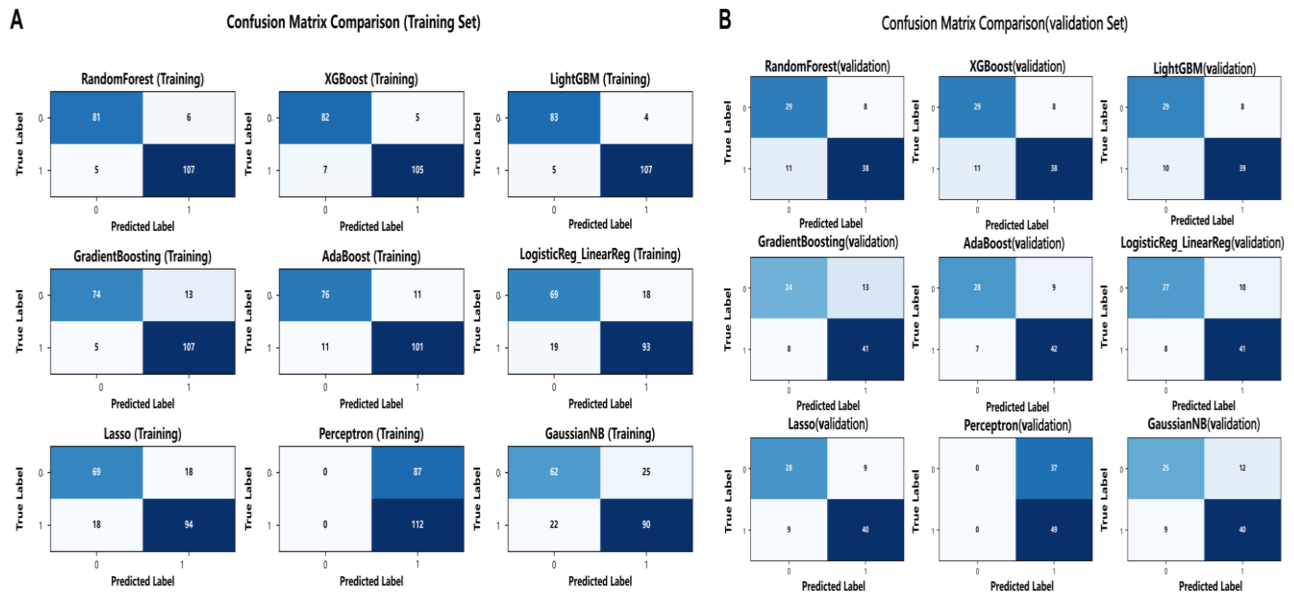

Figure S2 A comparison of confusion matrices for 8 ML models on both the training and validation sets. (A) displays the confusion matrices for the models evaluated on the training set, including RandomForest, XGBoost, LightGBM, GradientBoosting, AdaBoost, Logistic Regression, Lasso, and GaussianNB. The matrices show the true positive (TP), true negative (TN), false positive (FP), and false negative (FN) values for each model. The model's ability to correctly classify positive and negative samples is visually represented by the intensity of the dark blue squares (correct predictions) and light blue squares (incorrect predictions). (B) presents the confusion matrices for the same models on the validation set, providing a comparison of their generalizability.

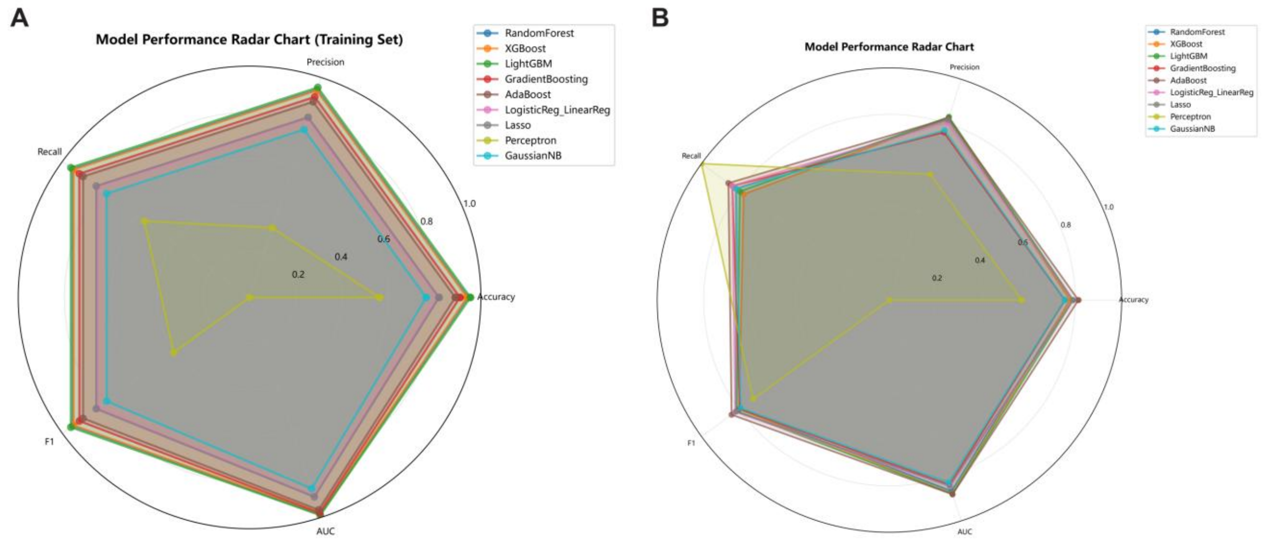

Figure S3 Precision–recall (PR) curves and related parameters of eight ML models. (A)PR-based performance metrics (sensitivity, specificity, precision, recall, F1 score) in the training cohort. (B) PR-based performance metrics in the validation cohort.

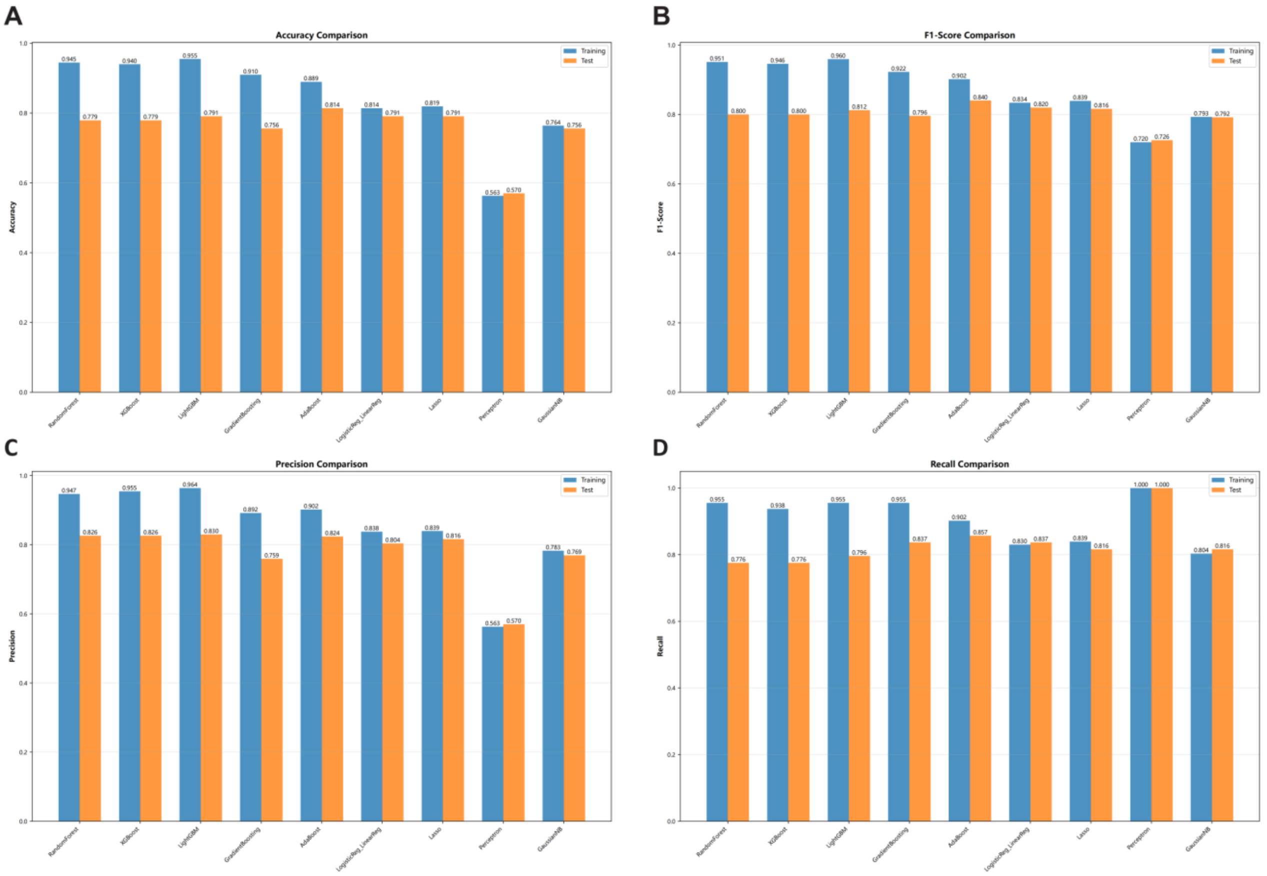

Figure S4 Performance evaluation of eight ML models on the training and test sets (A) Accuracy, (B) F1 Score, (C) Precision and (D) Recall.

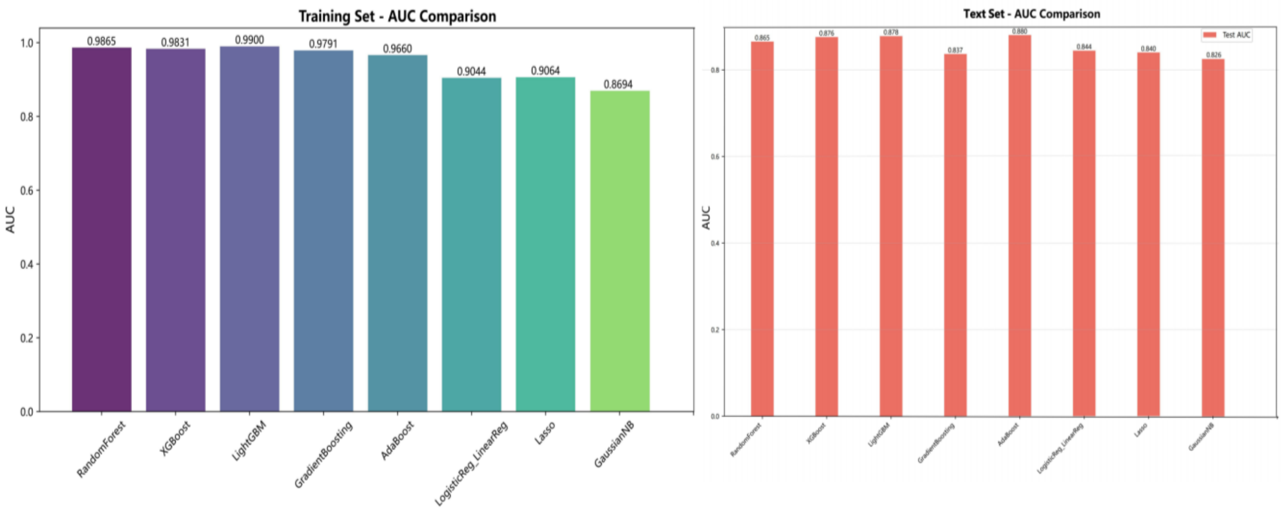

Figure S5 Comparison of AUC value distributions for eight ML models.
(A)AUC comparison in the training cohort; (B) AUC comparison in the validation cohort.

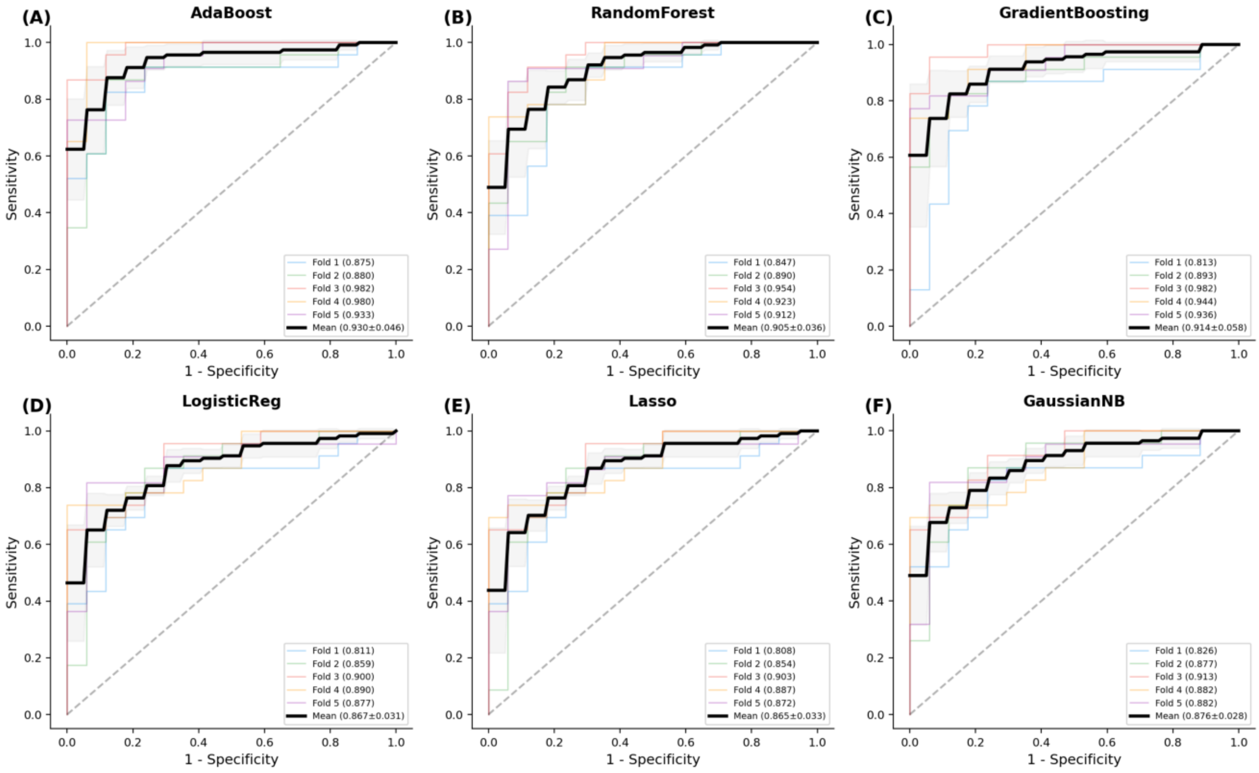

Figure S6 Five-fold stratified cross-validation ROC curves for each machine learning model (training cohort, n = 199). (A) AdaBoost (mean AUC = 0.930 ± 0.046), (B) RandomForest (0.905 ± 0.036), (C) GradientBoosting (0.913 ± 0.059), (D) LogisticReg (0.868 ± 0.032), (E) Lasso (0.865 ± 0.033), (F) GaussianNB (0.876 ± 0.028). Individual fold ROC curves (colored thin lines) and mean ROC curve (black bold line) with ±1 SD shading are shown. AdaBoost achieved the highest mean cross-validated AUC, confirming its

selection as the optimal model.

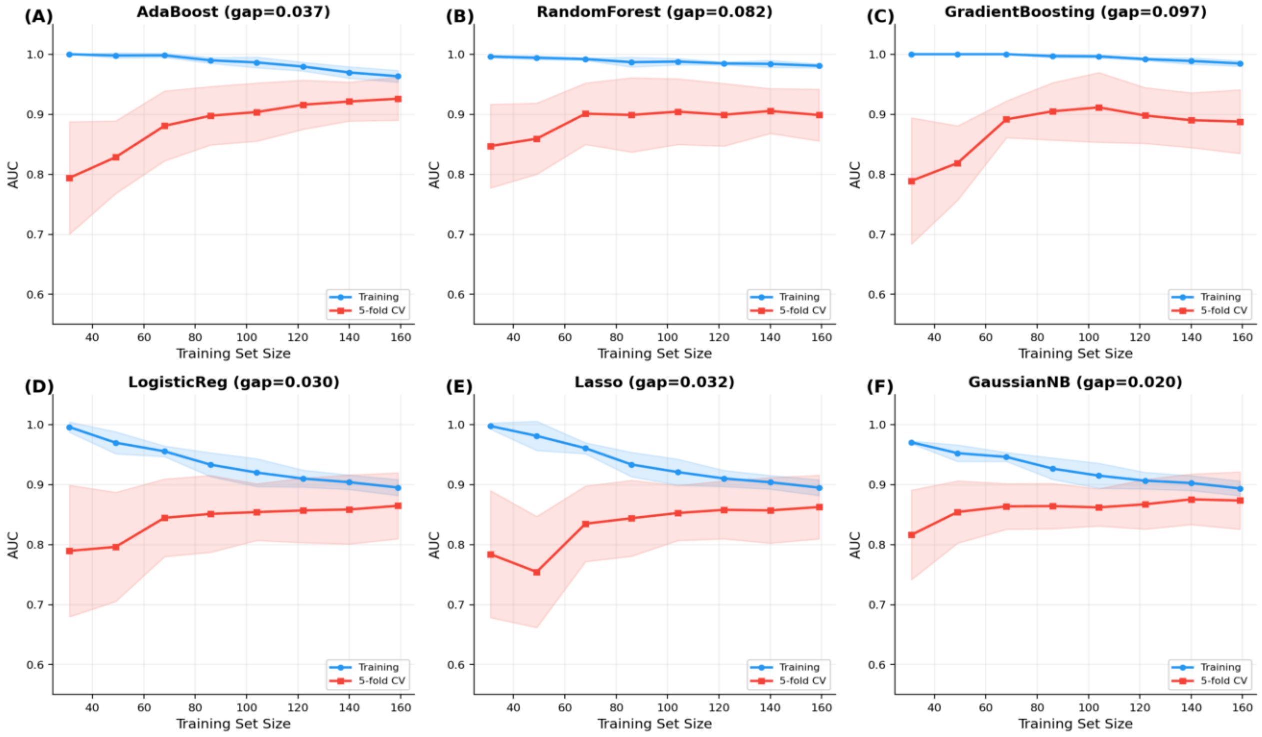

Figure S7 Learning curves for six machine learning models. (A) AdaBoost, (B) RandomForest, (C) GradientBoosting, (D) LogisticReg, (E) Lasso, (F) GaussianNB. Blue lines indicate training AUC; red lines indicate 5-fold cross-validation AUC. Shaded regions represent ±1 standard deviation. The training-validation gap at maximum sample size is shown in each title. AdaBoost (gap = 0.084) shows smaller gap than RandomForest (gap = 0.101) and GradientBoosting (gap = 0.096), supporting its superior generalizability.

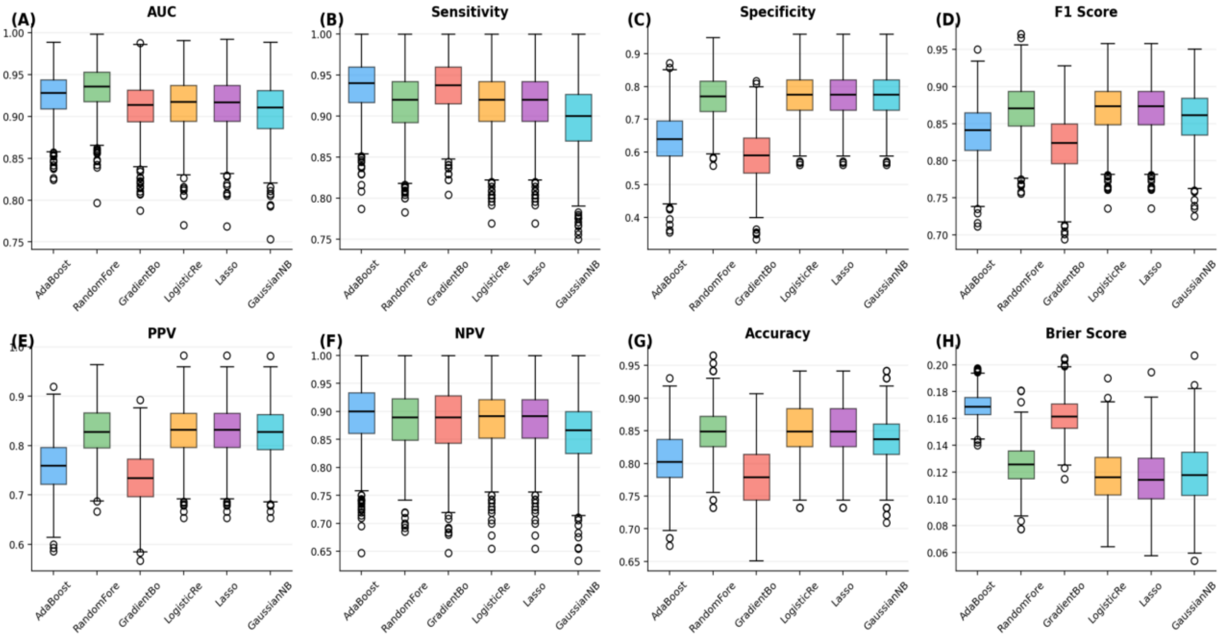

Figure S8 Bootstrap-derived 95% confidence interval distributions for all performance metrics in the validation cohort (n = 86; 1,000 bootstrap resamples). (A) AUC, (B) Sensitivity, (C) Specificity, (D) F1 Score, (E) PPV, (F) NPV, (G) Accuracy, (H) Brier Score. Box plots show the distribution of each metric across 1,000 bootstrap resamples for all six models. Boxes represent the interquartile range; whiskers extend to 1.5× IQR. These distributions quantify uncertainty in point estimates from the validation cohort.

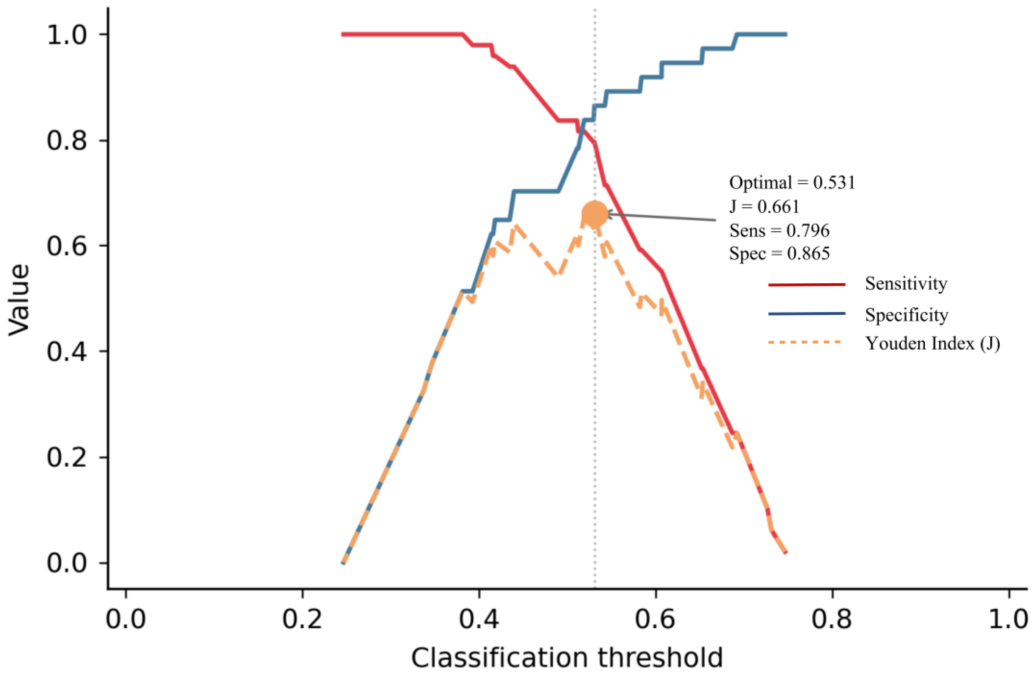

Figure S9 Youden-Index threshold optimization for the AdaBoost model in the validation cohort. Red:

sensitivity; blue: specificity; orange dashed: Youden Index (J). The optimal threshold is 0.531 (J = 0.661), yielding sensitivity = 0.796 and specificity = 0.865, compared with the default 0.5 threshold (sensitivity = 0.837, specificity = 0.784).

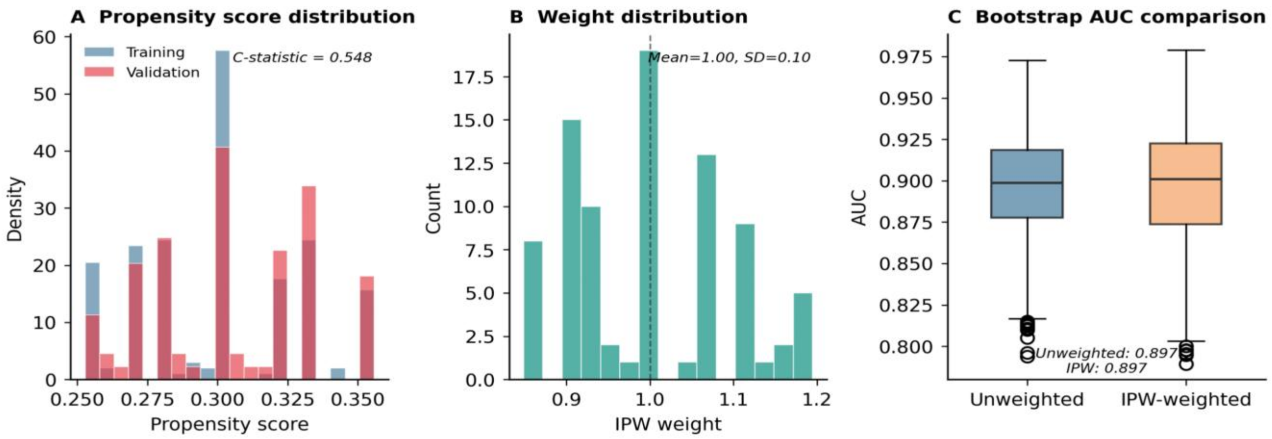

Figure S10 Inverse-probability-of-split-weighted (IPW) sensitivity analysis for the AdaBoost model. (A) Propensity score distributions for training (blue) and validation (red) cohorts; the propensity score model achieved a C-statistic of only 0.548. (B) Distribution of stabilized IPW weights for validation-set patients (mean = 1.00, SD = 0.12). (C) Bootstrap AUC comparison: unweighted AUC = 0.897 (95% CI: 0.825 – 0.957) versus IPW-weighted AUC = 0.897 (95% CI: 0.815 – 0.958), confirming that the three potentially imbalanced variables exert no meaningful influence on validation performance.

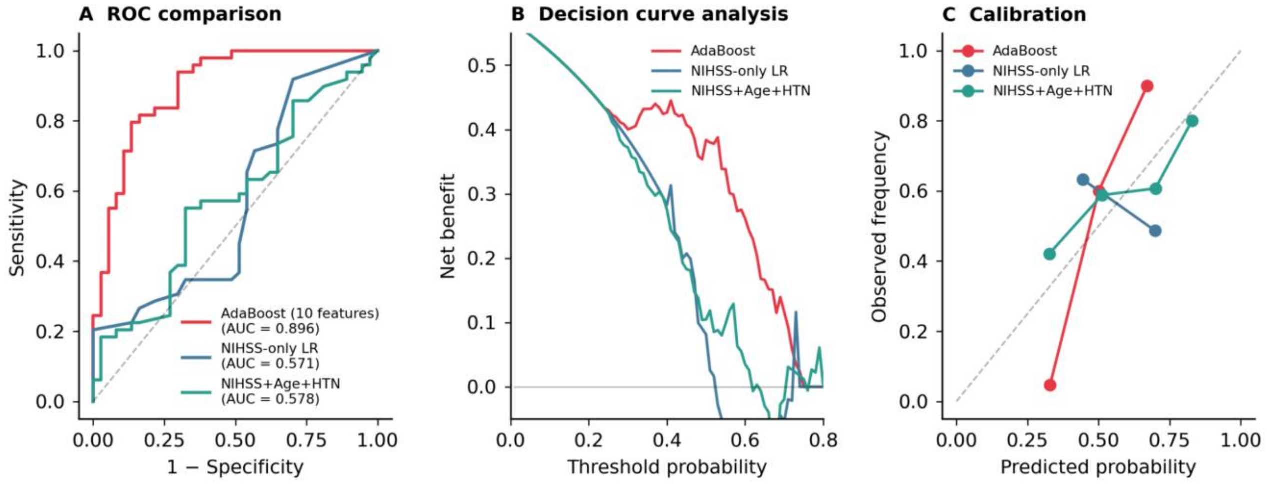

Figure S11 Comparison of AdaBoost (10 features) versus clinical baseline models in the validation cohort. (A) ROC curves: AdaBoost AUC = 0.896 vs. NIHSS-only LR AUC = 0.571 vs. NIHSS+Age+HTN composite AUC = 0.578. (B) Decision curve analysis: AdaBoost provides greater net clinical benefit across threshold probabilities 0.2 – 0.8. (C) Calibration comparison.

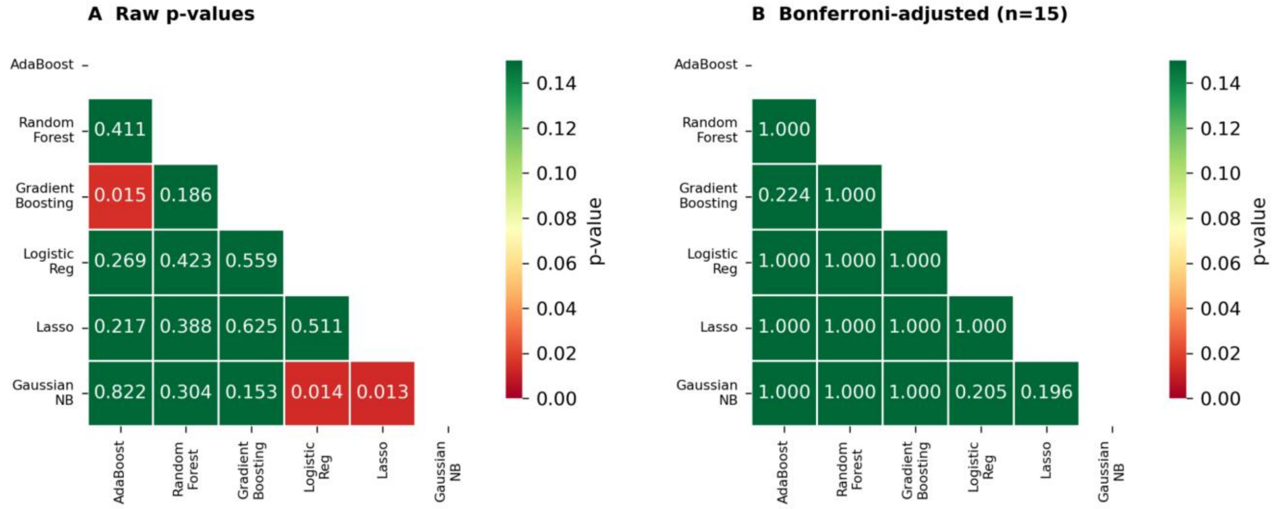

Figure S12 DeLong pairwise AUC comparison heatmaps for all model pairs in the validation cohort (n = 86). (A) Raw DeLong p-values. (B) Bonferroni-adjusted p-values for 15 pairwise comparisons (corrected α = 0.0033). After Bonferroni correction, no pairwise comparison reached statistical significance.

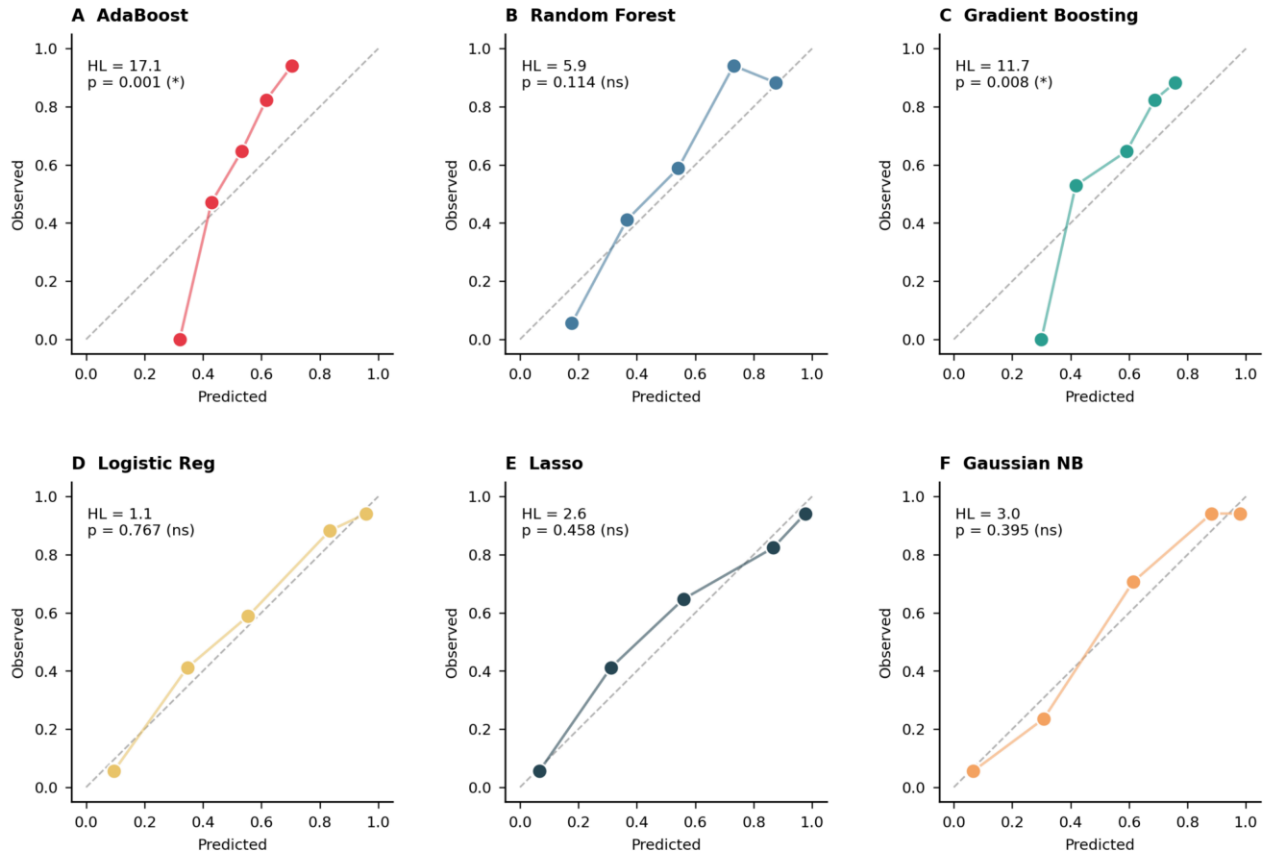

Figure S13 Hosmer－Lemeshow goodness-of-fit calibration plots for all six models in the validation cohort (n = 86). Each panel displays observed outcome frequency versus mean predicted probability, with the HL test

statistic and p-value annotated. Parametric models (Logistic Reg, Lasso, Gaussian NB) demonstrate acceptable calibration ($p > 0.05$), while ensemble models (AdaBoost, Gradient Boosting) show significant HL statistics.

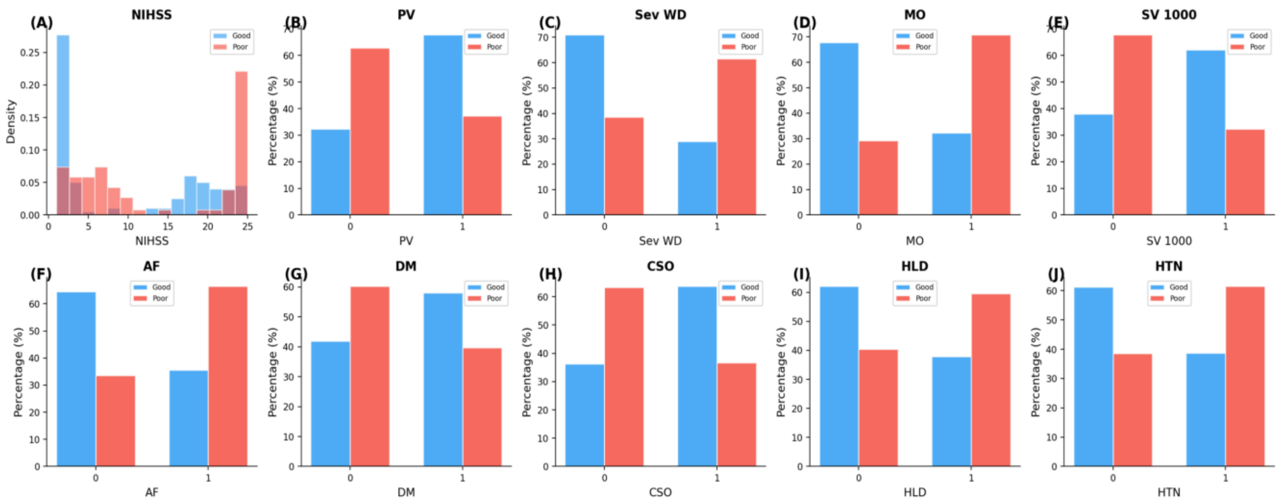

Figure S14 Distribution of 10 selected predictors stratified by outcome group. (A) NIHSS, (B) PV, (C) Sev WD, (D) MO, (E) SV 1000, (F) AF, (G) DM, (H) CSO, (I) HLD, (J) HTN. Binary variables are shown as grouped bar charts (percentage in each outcome group); the continuous variable NIHSS is shown as overlapping density histograms. Blue = good prognosis; red = poor prognosis. Clear separation is visible for AF, MO, PV, CSO, Sev WD, SV 1000, and NIHSS, consistent with their high Boruta importance scores.

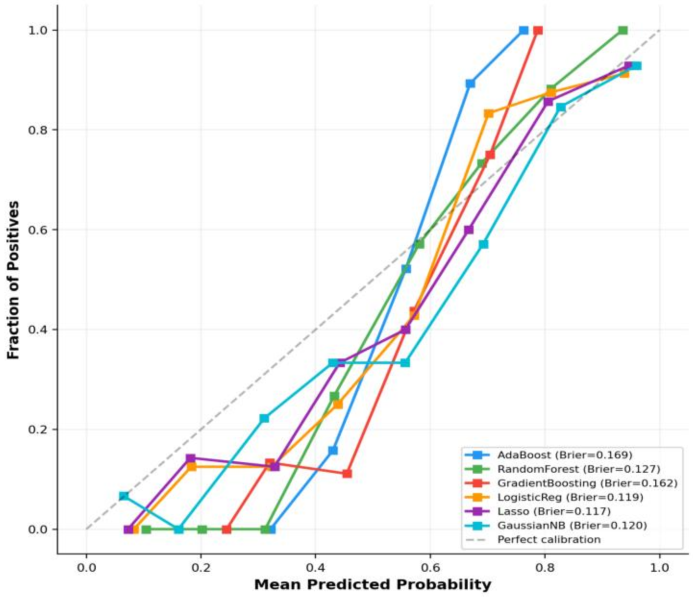

Figure S15 Calibration curves for all models in the validation cohort (n = 86). Calibration curves comparing predicted probabilities (x-axis) against observed event frequencies (y-axis) across decile groups for all six models. Brier scores are shown in the legend. The diagonal dashed line represents perfect calibration. AdaBoost demonstrates acceptable calibration with predicted probabilities aligned along the diagonal.

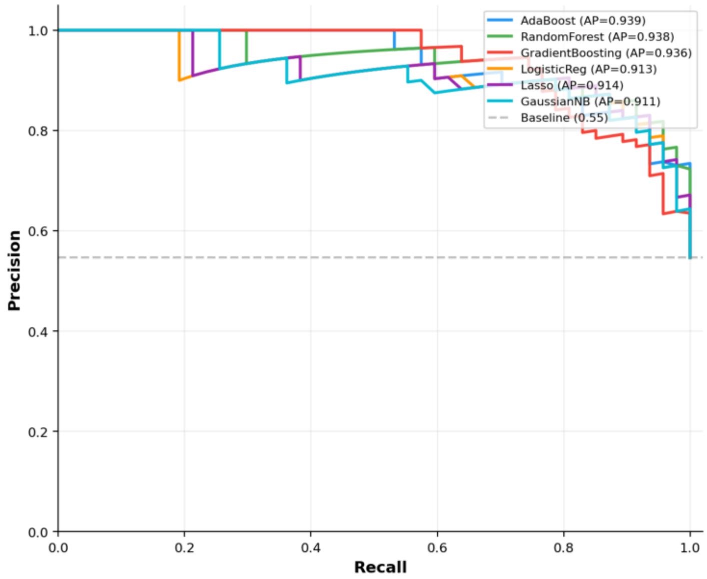

Figure S16 Precision-recall curves for all models in the validation cohort (n = 86). Precision-recall curves with average precision (AP) scores for all six models. The horizontal dashed line indicates baseline prevalence (56.49%). AP combines precision and recall into a single metric that accounts for class imbalance. AdaBoost (AP = 0.881) achieves competitive precision-recall performance.

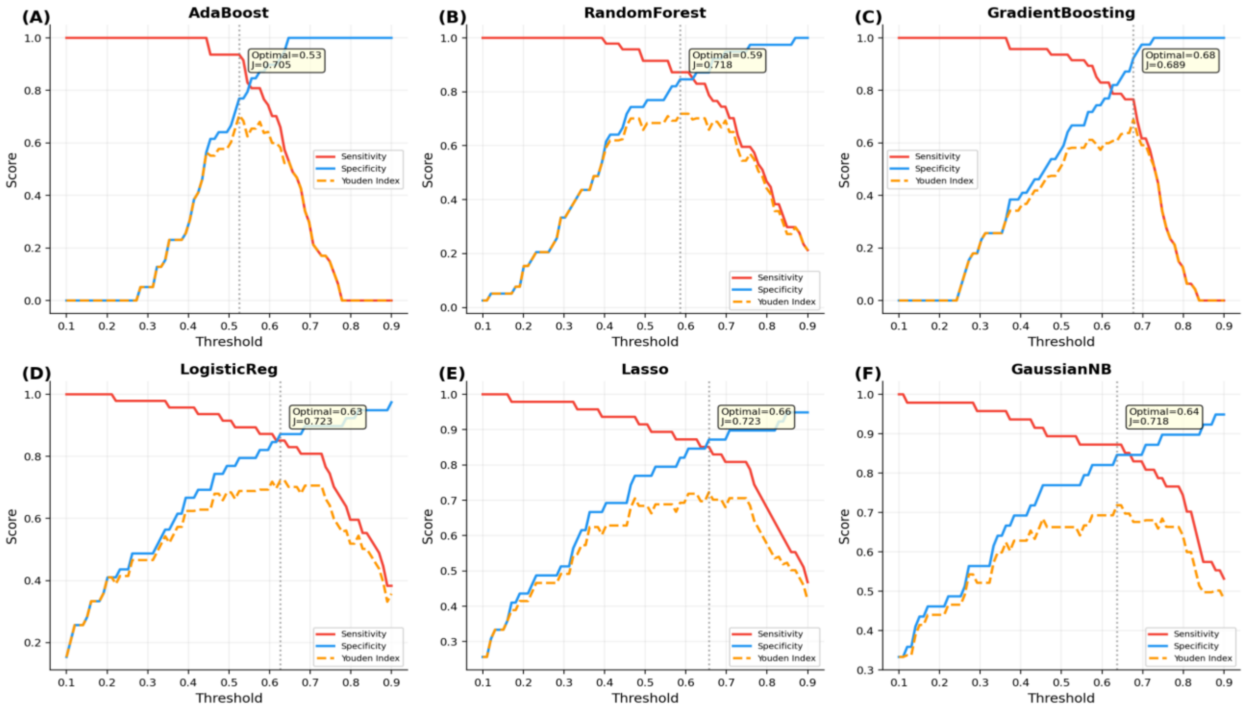

Figure S17 Threshold optimization via Youden Index for all models (validation cohort). (A) AdaBoost, (B) RandomForest, (C) GradientBoosting, (D) LogisticReg, (E) Lasso, (F) GaussianNB. Red lines show sensitivity; blue lines show specificity; orange dashed lines show the Youden Index (J = sensitivity + specificity − 1) across threshold values. The optimal threshold (vertical gray line) and corresponding Youden Index are annotated. Detailed optimal threshold values are provided in Table S21.

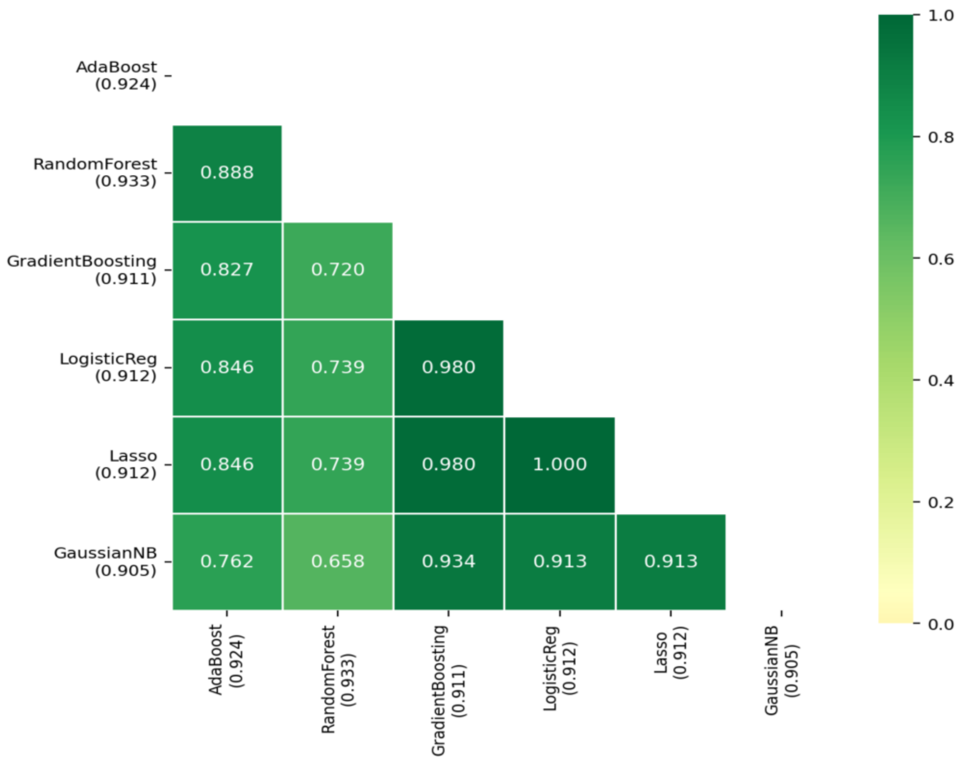

Figure S18 DeLong pairwise AUC comparison between all models (validation cohort). Lower-triangle heatmap showing DeLong test p-values for pairwise AUC comparisons. AUC values for each model are shown on the axes. Green cells indicate non-significant differences (p > 0.05); red cells indicate significant differences. Most top-performing models (AdaBoost, LightGBM, XGBoost) show non-significant pairwise differences, consistent with the limited statistical power of n = 86.

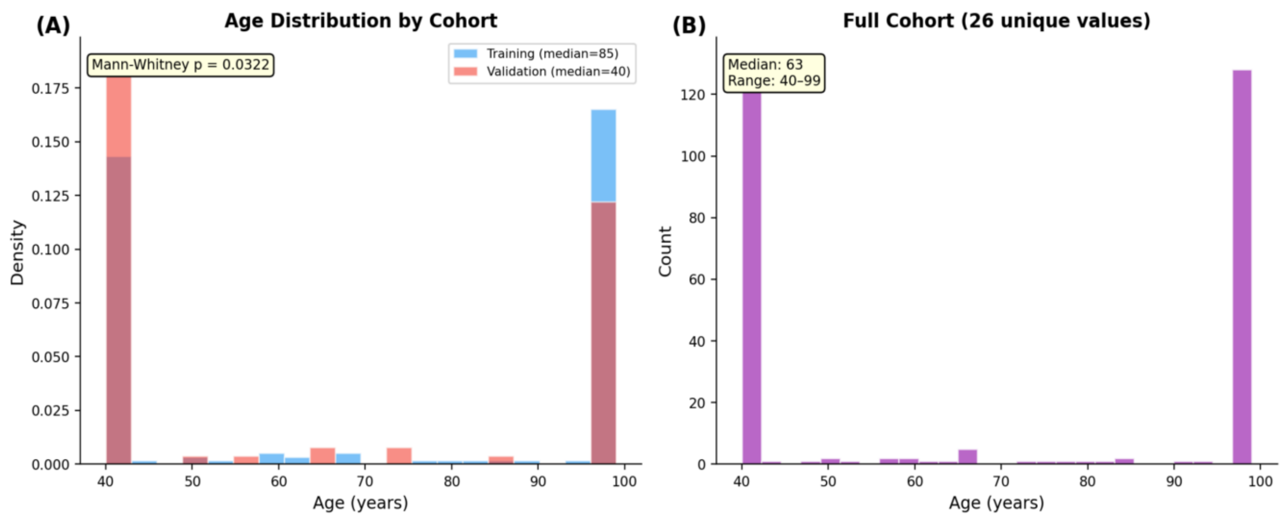

Figure S19 Age distribution analysis. (A) Age distributions in the training cohort (blue; median = 85) and validation cohort (red; median = 40). The Mann-Whitney U test confirms a significant difference (p = 0.032). (B)

Age distribution of the full cohort, showing 26 unique values with concentration at the extremes. This limited granularity likely reflects encoding conventions in the hospital electronic medical record system.

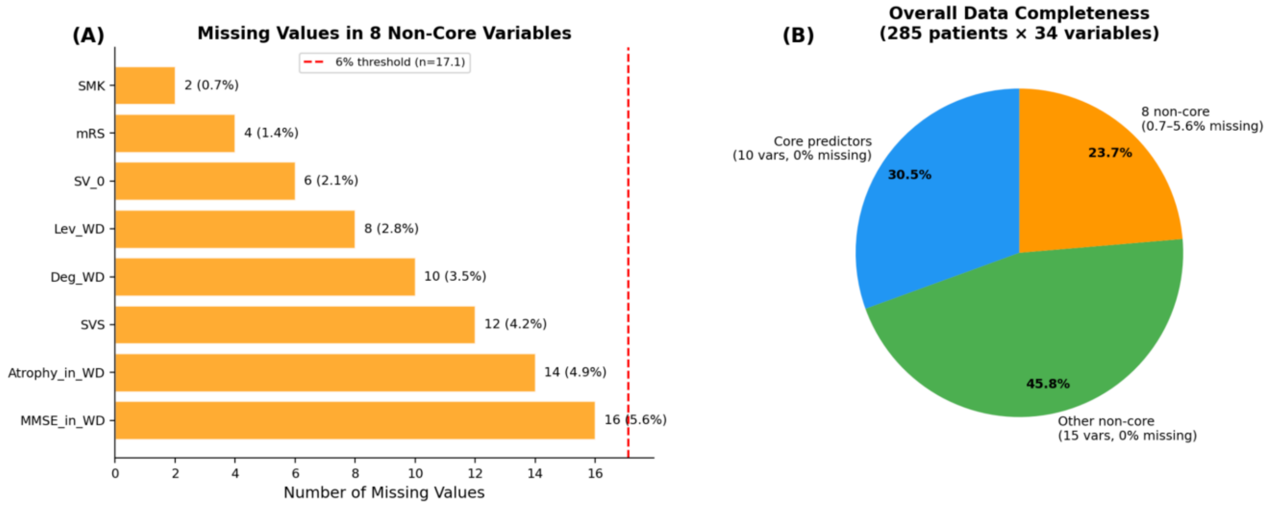

Figure S20 Missing data summary. (A) Number of missing values in 8 non-core variables with missing data (all ⩽6%). The red dashed line indicates the 6% threshold. MMSE had the highest missing rate (5.6%, n = 16), while smoking history had the lowest (0.7%, n = 2). None of these variables were selected by the Boruta algorithm. (B) Overall data completeness pie chart showing that core predictors (10 variables) and other non-core variables (15 variables) had 100% completeness.

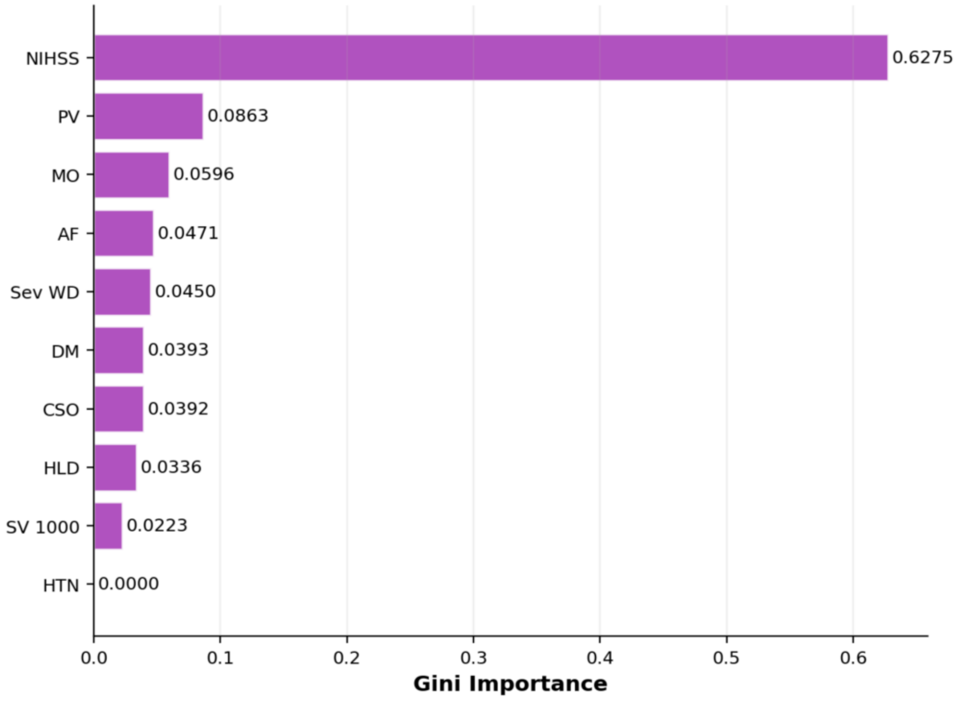

Figure S21 AdaBoost native feature importance (Gini importance). Horizontal bar chart showing Gini importance scores from the AdaBoost classifier. NIHSS shows the highest native importance, consistent with its top ranking in SHAP analysis (mean |SHAP| = 1.757). The ranking provides a complementary view to SHAP-based interpretations.

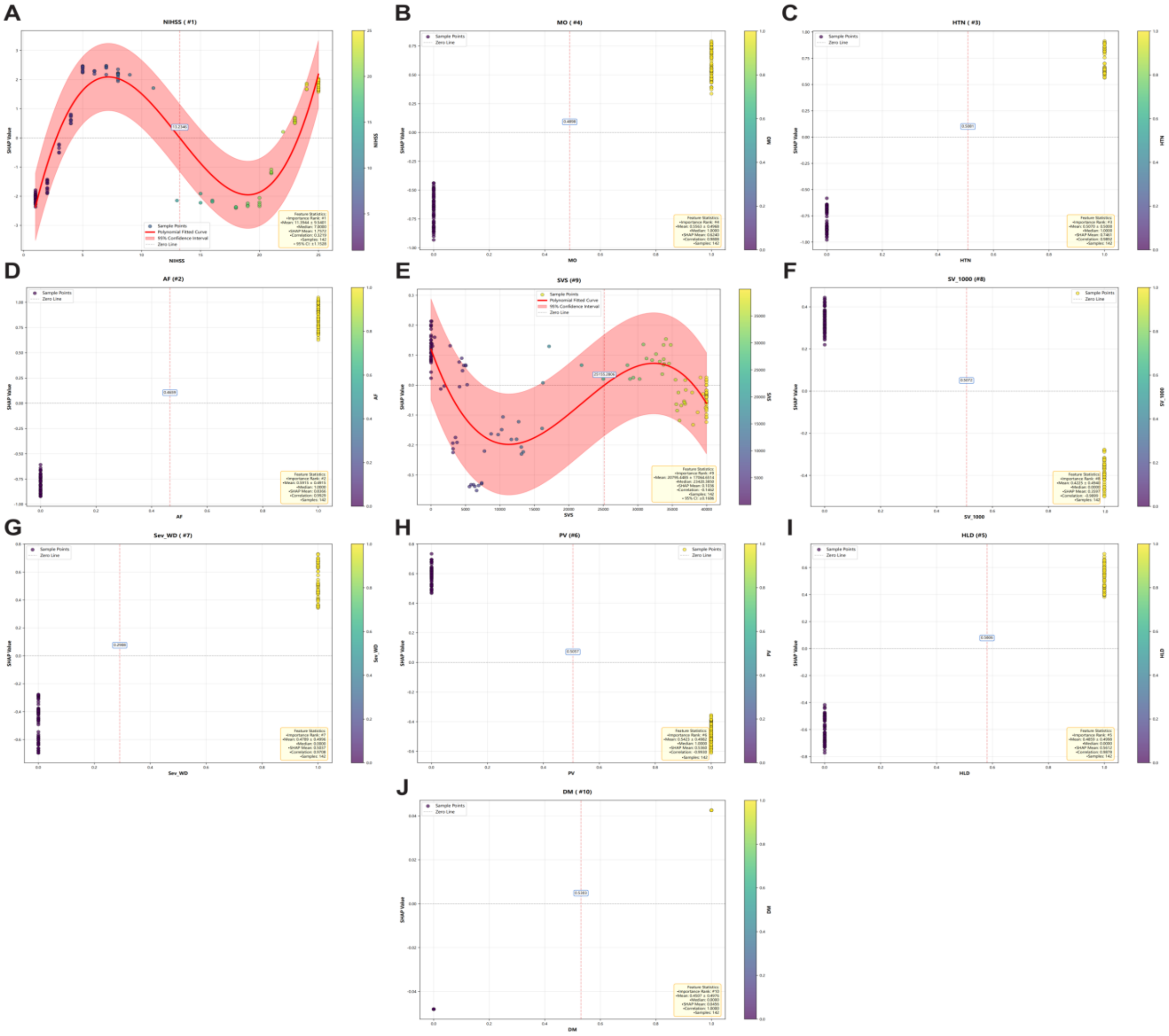

Figure S22 SHAP-based feature dependence plot. (A-J) Importance ranking of ten key features (NIHSS, AF, MO, etc.) on model prediction outcomes and their dependence on feature values.

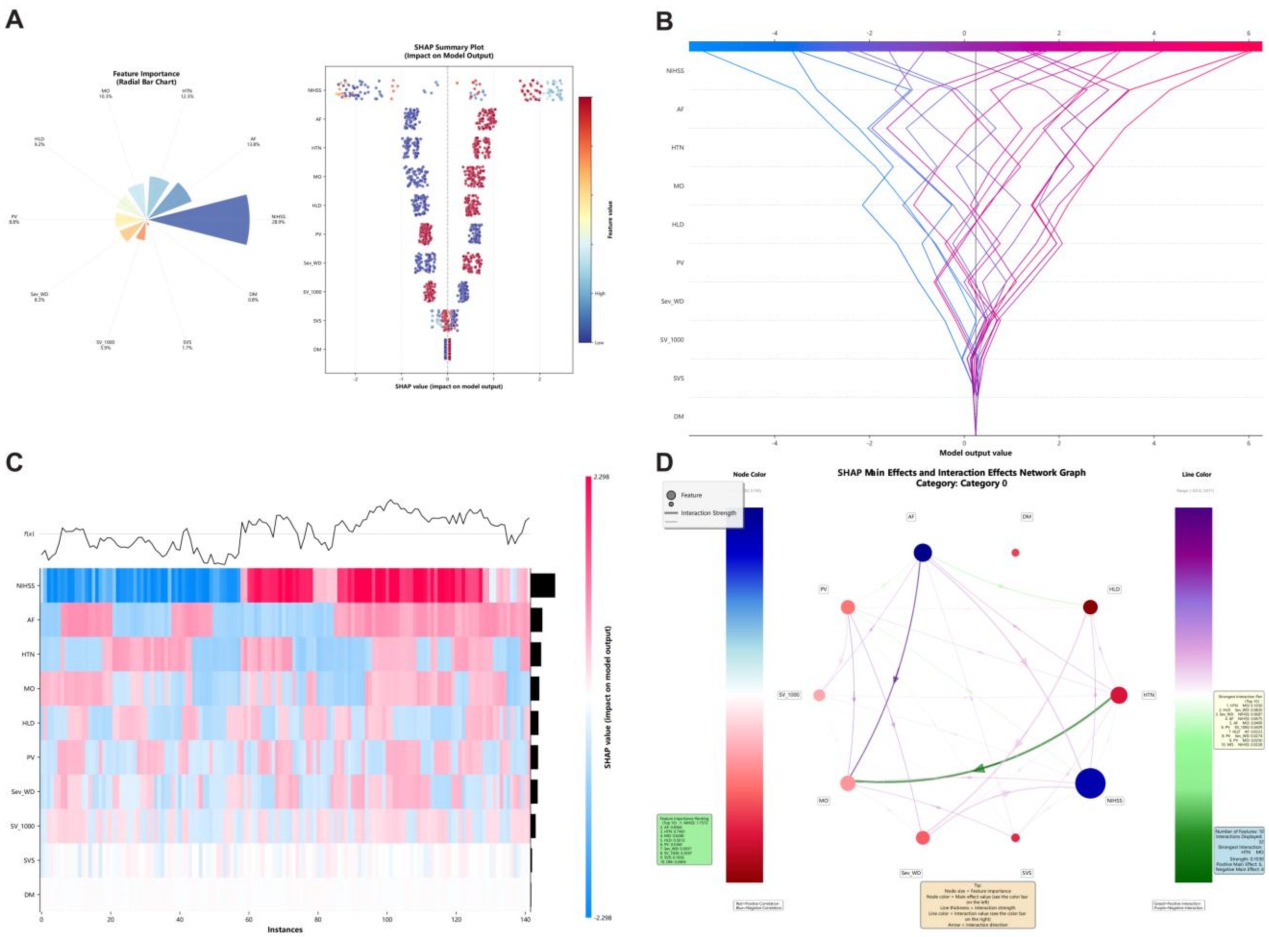

Figure S23 SHAP analysis reveals the decision mechanism of the optimal model.

(A) Summary plot: Feature importance and direction of impact; (B) Multi-sample decision plot: Prediction explanation for multiple samples, showing how each feature drives the model output from baseline to final prediction; (C) Heatmap: Contribution patterns across all samples; (D) Dependence plot: Feature effects and interactions.

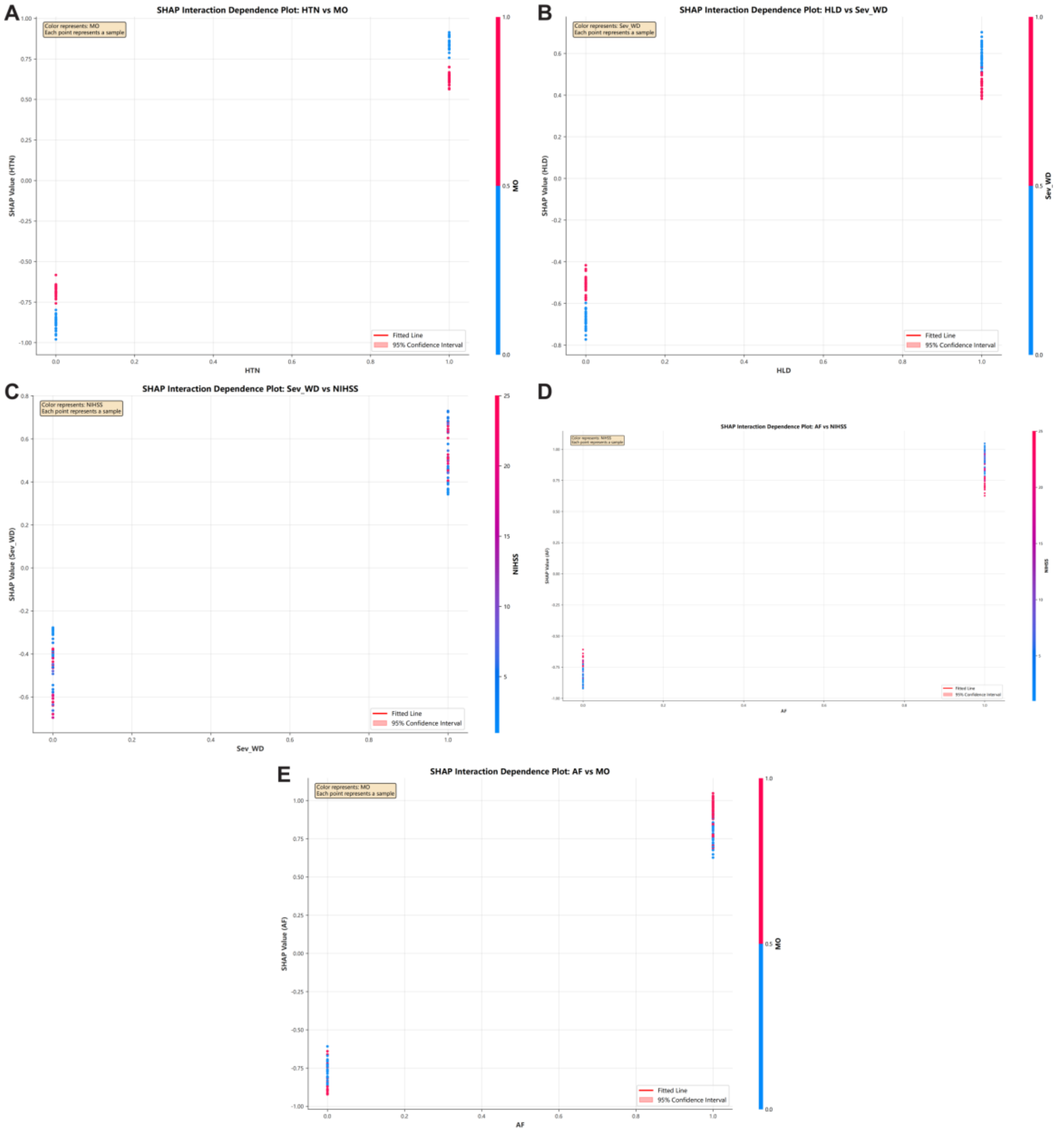

Figure S24 SHAP interaction plots of key features in the optimal model. (A) Interaction between HTN and MO on model prediction; (B) Interaction between HLD and Sev WD on model prediction; (C) Interaction between Sev WD and NIHSS on model prediction; (D) Interaction between AF and NIHSS on model prediction; (E) Interaction between AF and MO on model prediction.

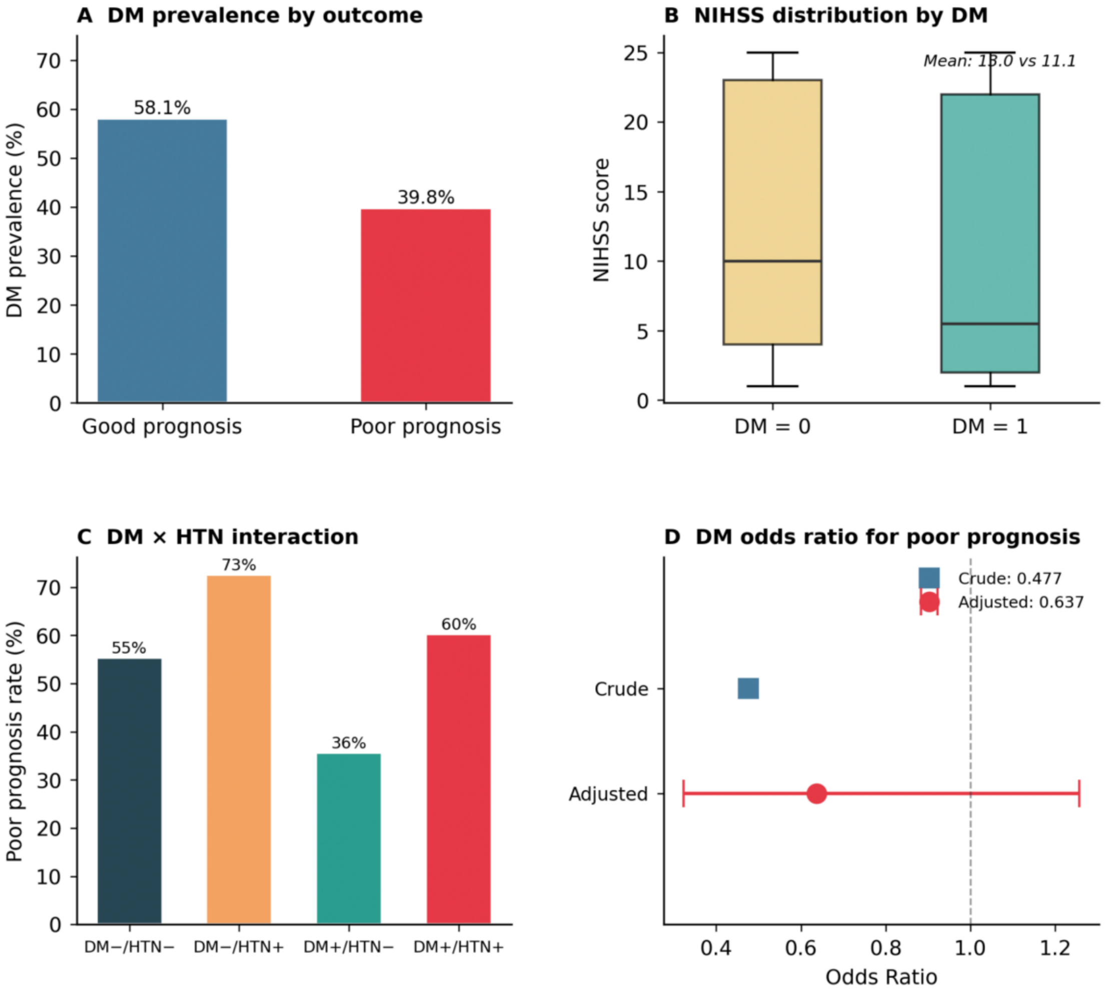

Figure S25 Diabetes mellitus paradox investigation. (A) DM prevalence by outcome group: DM is more prevalent in patients with good prognosis (58.1%) than poor prognosis (39.8%). (B) NIHSS distribution by DM status: diabetic patients show lower mean NIHSS (11.1 vs. 13.0). (C) DM × HTN interaction analysis on poor prognosis rates. (D) Forest plot showing crude OR = 0.477 and adjusted OR = 0.637 (95% CI: 0.323 – 1.256).
