# Supplementary material for: Development and validation of a machine learning model for predicting adverse prognosis in Wallerian degeneration patients based on clinical and imaging data
Source: Front Neurol. 2026 May 26;17:1840010. doi: 10.3389/fneur.2026.1840010 (PMC13246421; doi:10.3389/fneur.2026.1840010)
Supplement: Supplementary file 2 [file Supplementary_file_2.docx]

**Table S1 TRIPOD (Transparent Reporting of a multivariable prediction model for Individual Prognosis Or Diagnosis) checklist.**

| **Item** | **Section** | **Description** | **Reported** | **Location** |
| --- | --- | --- | --- | --- |
| 1 | Title | Identify as prediction model | Yes | Title |
| 2 | Abstract | Summary | Yes | Abstract (Revised) |
| 3a | Introduction | Context | Yes | Introduction |
| 3b | Introduction | Objectives | Yes | Intro Para 4 |
| 4a | Methods | Design | Yes | Methods Para 1 |
| 4b | Methods | Dates | Yes | Methods (Jan 2022-Jun 2024) |
| 5a | Participants | Setting | Yes | Methods Para 1 |
| 5b | Participants | Eligibility | Yes | Methods Para 2 |
| 6a | Outcome | Define outcome | Yes | Methods (Revised: mRS>2 at ≥3mo) |
| 6b | Outcome | Blind assessment | Yes | Methods (Revised: blinded readers) |
| 7a | Predictors | Define predictors | Yes | Methods (Revised: definitions) |
| 7b | Predictors | Blind assessment | Partial | Discussion |
| 8 | Sample size | Rationale | Yes | Methods; Discussion |
| 9 | Missing data | Handling | Yes | Methods (Revised); Table S2; Figure S20 |
| 10a | Statistical | Predictor handling | Yes | Methods Para 4-5 |
| 10b | Statistical | Model building | Yes | Methods; Figure S1 |
| 10c | Statistical | Internal validation | Yes | Methods (5-fold CV, bootstrap) |
| 10d | Statistical | Performance measures | Yes | Methods Para 5-6 |
| 10e | Statistical | Model comparison | Yes | DeLong test; Table 4 |
| 11 | Risk groups | Creation | Yes | Methods Para 3 |
| 13a | Flow | Participant flow | Yes | Figure 1 |
| 13b | Demographics | Characteristics | Yes | Table 1; S4 |
| 14a | Development | N and events | Yes | Results Para 1 |
| 14b | Development | Unadjusted | Yes | Table S4 |
| 15a | Specification | Full model | Yes | Figure 3; Table 2 |
| 15b | Specification | Model use | Yes | Discussion |
| 16 | Performance | Metrics+CIs | Yes | Tables S11,S13,S20; Figs S8,S15-S16 |
| 17 | Updating | Model updating | nan | — |
| 18 | Limitations | Limitations | Yes | Discussion (Revised) |
| 19a | Implications | Clinical use | Yes | Discussion (Revised) |
| 19b | Implications | Updating potential | Yes | Discussion |
| 20 | Supplementary | Availability | Yes | Data Availability |

**Table S2 Missing data summary for all 36 candidate variables. Eight non-core independent variables with missing rates ≤6% were identified; none were selected by the Boruta algorithm.**

| **Variable** | **Description** | **Category** | **N** | **N-Missing** | **Missing-Rate** | **Boruta** |
| --- | --- | --- | --- | --- | --- | --- |
| NIHSS | NIHSS score | Core predictor (selected) | 285 | 0 | 0.0% | Yes |
| HTN | Hypertension | Core predictor (selected) | 285 | 0 | 0.0% | Yes |
| HLD | Hyperlipidemia | Core predictor (selected) | 285 | 0 | 0.0% | Yes |
| DM | Diabetes | Core predictor (selected) | 285 | 0 | 0.0% | Yes |
| AF | Atrial Fibrillation | Core predictor (selected) | 285 | 0 | 0.0% | Yes |
| PV | Periventricular | Core predictor (selected) | 285 | 0 | 0.0% | Yes |
| MO | Medulla Oblongata | Core predictor (selected) | 285 | 0 | 0.0% | Yes |
| CSO | Centrum Semiovale | Core predictor (selected) | 285 | 0 | 0.0% | Yes |
| SV-1000 | Subcortical Vol >1000 | Core predictor (selected) | 285 | 0 | 0.0% | Yes |
| Sev-WD | Severity of WD | Core predictor (selected) | 285 | 0 | 0.0% | Yes |
| Type-stroke | Stroke type | Non-core (complete) | 285 | 0 | 0.0% | No (rejected) |
| Time-WD | Time since WD | Non-core (complete) | 285 | 0 | 0.0% | No (rejected) |
| Sex | Sex | Non-core (complete) | 285 | 0 | 0.0% | No (rejected) |
| Age | Age | Non-core (complete) | 285 | 0 | 0.0% | No (rejected) |
| TL | Temporal Lobe | Non-core (complete) | 285 | 0 | 0.0% | No (rejected) |
| BG | Basal Ganglia | Non-core (complete) | 285 | 0 | 0.0% | No (rejected) |
| FL | Frontal Lobe | Non-core (complete) | 285 | 0 | 0.0% | No (rejected) |
| PL | Parietal Lobe | Non-core (complete) | 285 | 0 | 0.0% | No (rejected) |
| OL | Occipital Lobe | Non-core (complete) | 285 | 0 | 0.0% | No (rejected) |
| MCA | MCA | Non-core (complete) | 285 | 0 | 0.0% | No (rejected) |
| ACA | ACA | Non-core (complete) | 285 | 0 | 0.0% | No (rejected) |
| PCA | PCA | Non-core (complete) | 285 | 0 | 0.0% | No (rejected) |
| PC | Post. Circulation | Non-core (complete) | 285 | 0 | 0.0% | No (rejected) |
| LLP | Left Limb Paresis | Non-core (complete) | 285 | 0 | 0.0% | No (rejected) |
| UPP | Upper Limb Paresis | Non-core (complete) | 285 | 0 | 0.0% | No (rejected) |
| MMSE-in-WD | Mini-Mental State Examination | Non-core (with missing) | 285 | 16 | 5.6% | No (rejected) |
| Atrophy-in-WD | Asymmetry Index of WD (AIWD) | Non-core (with missing) | 285 | 14 | 4.9% | No (rejected) |
| SVS | Signs and Symptoms score | Non-core (with missing) | 285 | 12 | 4.2% | No (rejected) |
| Deg-WD | Degree of WD | Non-core (with missing) | 285 | 10 | 3.5% | No (rejected) |
| Lev-WD | Level of WD involvement | Non-core (with missing) | 285 | 8 | 2.8% | No (rejected) |
| SV-0 | Subcortical Volume baseline | Non-core (with missing) | 285 | 6 | 2.1% | No (rejected) |
| MRS | Baseline mRS at WD confirmation | Non-core (with missing) | 285 | 4 | 1.4% | No (rejected) |
| SMK | Smoking history | Non-core (with missing) | 285 | 2 | 0.7% | No (rejected) |
| WD | Poor prognosis (outcome) | Outcome | 285 | 0 | 0.0% | nan |

**Table S3 AIC and BIC values for backward stepwise logistic regression model selection. The full 10-variable model had the lowest AIC (187.9); removing any variable increased AIC, justifying retention of all features including those with borderline p-values.**

| **Step** | **Action** | **N** | **AIC** | **BIC** | **LL** | **ΔAIC** |
| --- | --- | --- | --- | --- | --- | --- |
| 0 | Full model (all 10 features) | 10 | 187.9 | 224.1 | -82.9 | nan |
| 1 | Remove NIHSS | 9 | 189.2 | 222.1 | -84.6 | 1.3 |
| 2 | Remove PV | 9 | 202.7 | 235.7 | -91.4 | 14.8 |
| 3 | Remove Sev WD | 9 | 198 | 231 | -89 | 10.1 |
| 4 | Remove MO | 9 | 195 | 227.9 | -87.5 | 7.1 |
| 5 | Remove SV 1000 | 9 | 193.9 | 226.8 | -86.9 | 6 |
| 6 | Remove AF | 9 | 197 | 230 | -88.5 | 9.1 |
| 7 | Remove DM | 9 | 190.5 | 223.5 | -85.3 | 2.7 |
| 8 | Remove CSO | 9 | 190.1 | 223 | -85.1 | 2.2 |
| 9 | Remove HLD | 9 | 189.5 | 222.4 | -84.7 | 1.6 |
| 10 | Remove HTN | 9 | 189.5 | 222.4 | -84.7 | 1.6 |

**Table S4 Comparison of baseline clinical and imaging characteristics between patients with poor and favorable prognosis of WD.**

|  | **[ALL]**  **N=285** | **No N=124** | **Yes N=161** | **p.overall** |
| --- | --- | --- | --- | --- |
| **Type stroke, n (%)** |  |  |  | 0.287 |
| NHS | 147 (51.58%) | 59 (47.58%) | 88 (54.66%) |  |
| HS | 138 (48.42%) | 65 (52.42%) | 73 (45.34%) |  |
| **Time WD, n (%)** |  |  |  | 0.673 |
| ≤ 2 months | 135 (47.37%) | 61 (49.19%) | 74 (45.96%) |  |
| > 3 months | 150 (52.63%) | 63 (50.81%) | 87 (54.04%) |  |
| **Sex, n (%)** |  |  |  | 0.248 |
| Female | 128 (44.91%) | 61 (49.19%) | 67 (41.61%) |  |
| Male | 157 (55.09%) | 63 (50.81%) | 94 (58.39%) |  |
| **Age** | 63.00  [40.00;99.00] | 42.50  [40.00;99.00] | 73.00  [40.00;99.00] | 0.303 |
| **MRS＞2, n (%)** |  |  |  | 0.489 |
| No | 137 (48.07%) | 63 (50.81%) | 74 (45.96%) |  |
| Yes | 148 (51.93%) | 61 (49.19%) | 87 (54.04%) |  |
| **Hypertension, n (%)** |  |  |  | <0.001 |
| No | 138 (48.42%) | 76 (61.29%) | 62 (38.51%) |  |
| Yes | 147 (51.58%) | 48 (38.71%) | 99 (61.49%) |  |
| **Smoking, n (%)** |  |  |  | 0.980 |
| No | 148 (51.93%) | 65 (52.42%) | 83 (51.55%) |  |
| Yes | 137 (48.07%) | 59 (47.58%) | 78 (48.45%) |  |
| **Hyperlipidemia, n (%)** |  |  |  | <0.001 |
| No | 142 (49.82%) | 77 (62.10%) | 65 (40.37%) |  |
| Yes | 143 (50.18%) | 47 (37.90%) | 96 (59.63%) |  |
| **Diabetes Mellitus, n (%)** |  |  |  | 0.003 |
| No | 149 (52.28%) | 52 (41.94%) | 97 (60.25%) |  |
| Yes | 136 (47.72%) | 72 (58.06%) | 64 (39.75%) |  |
| **Atrial Fibrillation, n (%)** |  |  |  | <0.001 |
| No | 134 (47.02%) | 80 (64.52%) | 54 (33.54%) |  |
| Yes | 151 (52.98%) | 44 (35.48%) | 107 (66.46%) |  |
| **PV, n (%)** |  |  |  | <0.001 |
| No | 141 (49.47%) | 40 (32.26%) | 101 (62.73%) |  |
| Yes | 144 (50.53%) | 84 (67.74%) | 60 (37.27%) |  |
| **TL, n (%)** |  |  |  | 0.774 |
| No | 151 (52.98%) | 64 (51.61%) | 87 (54.04%) |  |
| Yes | 134 (47.02%) | 60 (48.39%) | 74 (45.96%) |  |
| **BG, n (%)** |  |  |  | 0.102 |
| No | 141 (49.47%) | 54 (43.55%) | 87 (54.04%) |  |
| Yes | 144 (50.53%) | 70 (56.45%) | 74 (45.96%) |  |
| **FL, n (%)** |  |  |  | 0.457 |
| No | 148 (51.93%) | 68 (54.84%) | 80 (49.69%) |  |
| Yes | 137 (48.07%) | 56 (45.16%) | 81 (50.31%) |  |
| **PL, n (%)** |  |  |  | 0.659 |
| No | 144 (50.53%) | 65 (52.42%) | 79 (49.07%) |  |
| Yes | 141 (49.47%) | 59 (47.58%) | 82 (50.93%) |  |
| **OL, n (%)** |  |  |  | 0.358 |
| No | 141 (49.47%) | 57 (45.97%) | 84 (52.17%) |  |
| Yes | 144 (50.53%) | 67 (54.03%) | 77 (47.83%) |  |
| **CSO, n (%)** |  |  |  | <0.001 |
| No | 147 (51.58%) | 45 (36.29%) | 102 (63.35%) |  |
| Yes | 138 (48.42%) | 79 (63.71%) | 59 (36.65%) |  |
| **SV 1000, n (%)** |  |  |  | <0.001 |
| No | 156 (54.74%) | 47 (37.90%) | 109 (67.70%) |  |
| Yes | 129 (45.26%) | 77 (62.10%) | 52 (32.30%) |  |
| **SV 0, n (%)** |  |  |  | 0.150 |
| No | 146 (51.23%) | 57 (45.97%) | 89 (55.28%) |  |
| Yes | 139 (48.77%) | 67 (54.03%) | 72 (44.72%) |  |
| **MCA, n (%)** |  |  |  | 0.003 |
| No | 156 (54.74%) | 55 (44.35%) | 101 (62.73%) |  |
| Yes | 129 (45.26%) | 69 (55.65%) | 60 (37.27%) |  |
| **ACA, n (%)** |  |  |  | 0.681 |
| No | 142 (49.82%) | 64 (51.61%) | 78 (48.45%) |  |
| Yes | 143 (50.18%) | 60 (48.39%) | 83 (51.55%) |  |
| **PCA, n (%)** |  |  |  | 0.463 |
| No | 130 (45.61%) | 53 (42.74%) | 77 (47.83%) |  |
| Yes | 155 (54.39%) | 71 (57.26%) | 84 (52.17%) |  |
| **PC, n (%)** |  |  |  | 0.673 |
| No | 135 (47.37%) | 61 (49.19%) | 74 (45.96%) |  |
| Yes | 150 (52.63%) | 63 (50.81%) | 87 (54.04%) |  |
| **LLP, n (%)** |  |  |  | 0.651 |
| No | 148 (51.93%) | 62 (50.00%) | 86 (53.42%) |  |
| Yes | 137 (48.07%) | 62 (50.00%) | 75 (46.58%) |  |
| **UPP, n (%)** |  |  |  | 0.673 |
| No | 135 (47.37%) | 61 (49.19%) | 74 (45.96%) |  |
| Yes | 150 (52.63%) | 63 (50.81%) | 87 (54.04%) |  |
| **MO, n (%)** |  |  |  | <0.001 |
| No | 131 (45.96%) | 84 (67.74%) | 47 (29.19%) |  |
| Yes | 154 (54.04%) | 40 (32.26%) | 114 (70.81%) |  |
| **Sev WD, n (%)** |  |  |  | <0.001 |
| No | 150 (52.63%) | 88 (70.97%) | 62 (38.51%) |  |
| Yes | 135 (47.37%) | 36 (29.03%) | 99 (61.49%) |  |
| **SVS (%)** | 13379.46  [824.96;38393.11] | 6762.42  [59.20;35459.23] | 30985.69  [2615.04;39948.63] | 0.005 |
| **Lev WD** | 3.00  [1.00;15.00] | 1.00  [1.00;15.00] | 7.00  [1.00;15.00] | 0.189 |
| **Deg WD** | 0.48  [0.05;0.66] | 0.66  [0.05;0.66] | 0.30  [0.05;0.66] | 0.058 |
| **MMSE** | 20.00  [17.00;29.00] | 19.00  [17.00;29.00] | 21.00  [18.00;29.00] | 0.072 |
| **AIWD** | 0.14  [0.03;0.43] | 0.12  [0.03;0.41] | 0.18  [0.04;0.44] | 0.088 |
| **NIHSS** | 8.00  [2.00;23.00] | 4.00  [1.00;19.00] | 9.00  [5.00;25.00] | <0.001 |

Abbreviations: Type Stroke, Type of Stroke; HS, Hemorrhagic Stroke; NHS, Non-hemorrhagic Stroke; Time WD, Time since diagnosis of WD; MRS>2, Modified Rankin Scale>2; PV, Periventricular ; TL, Temporal Lobe; BG, Basal Ganglia; FL, Frontal Lobe; PL, Parietal Lobe; OL, Occipital Lobe; CSO, Centrum Semiovale; SV 1000/SV 0, Subcortical Volume [1000/0]; MCA, Middle Cerebral Artery; ACA, Anterior Cerebral Artery; PCA, Posterior Cerebral Artery; PC, Posterior Circulation; LLP, Left Limb Paresis; UPP, Upper Limb Paresis; MO, Medulla Oblongata; Sev WD, Severity of WD; SVS, Signs and Symptoms; Lev WD ,Level of WD; Deg WD, Degree of WD; AIWD, Asymmetry Index of Wallerian Degeneration.

**Table S5 The importance metrics of each variable in the Boruta algorithm, including mean importance (meanImp), median importance (medianImp), minimum importance (minImp), maximum importance (maxImp), and the frequency of being selected as important (normHits).**

|  | **MeanImp** | **MedianImp** | **MinImp** | **MaxImp** | **NormHits** |
| --- | --- | --- | --- | --- | --- |
| Type stroke | 0.32663721 | 0.27009605 | -0.5160005 | 2.2856506 | 0 |
| Time WD | -0.27949111 | -0.23508621 | -1.9806223 | 1.3072522 | 0 |
| Sex | -0.42561312 | -0.51493511 | -2.3413445 | 1.395065 | 0 |
| MRS | -0.36722557 | -0.46619884 | -1.6807554 | 0.7667051 | 0 |
| HTN | 2.10415446 | 2.05100455 | -0.7098094 | 4.7174356 | 0.43434343 |
| SMK | -0.16127671 | -0.02981825 | -1.4385816 | 1.0558887 | 0 |
| HLD | 3.14483560 | 3.13372088 | 0.511037 | 5.3660176 | 0.72727273 |
| DM | 4.94551250 | 4.91790615 | 2.380408 | 7.4688473 | 0.94949495 |
| AF | 7.56085814 | 7.61502890 | 4.694180 | 10.030592 | 1 |
| PV | 12.46023749 | 12.51957417 | 9.5783032 | 15.962476 | 1 |
| TL | -0.83598263 | -1.25373934 | -2.3629076 | 1.0739765 | 0 |
| BG | 0.02962144 | 0.02356899 | -1.4078257 | 1.8700781 | 0 |
| FL | -0.84784907 | -0.64201172 | -2.7820904 | 0.7876166 | 0 |
| PL | 0.28112253 | -0.11349923 | -0.8739988 | 1.9311301 | 0 |
| OL | 0.08237042 | 0.25304257 | -1.4348353 | 0.9366919 | 0 |
| CSO | 3.70022242 | 3.73242341 | 1.1694002 | 6.2326469 | 0.70707071 |
| SV 1000 | 7.67764434 | 7.78991549 | 4.3330368 | 11.637765 | 1 |
| SV 0 | -0.26467461 | -0.09664246 | -2.5963003 | 1.1449356 | 0 |
| MCA | 0.74112122 | 0.68907916 | -1.4071659 | 2.6760726 | 0.01010101 |
| ACA | -0.66845888 | -0.3369468 | -2.4624443 | 1.142491 | 0 |
| PCA | -0.22146453 | -0.28555771 | -1.39345 | 0.9136064 | 0 |
| PC | -0.18138043 | -0.25320609 | -2.1155255 | 1.68426 | 0 |
| LLP | -0.05765362 | -0.11642944 | -2.104414 | 2.1728413 | 0 |
| UPP | -0.51732123 | -0.82692822 | -1.6693165 | 1.1744272 | 0 |
| MO | 8.00118676 | 8.1141672 | 4.4414679 | 11.2239691 | 0.989899 |
| Sev WD | 10.0851069 | 9.95200825 | 6.4028859 | 13.3335832 | 1 |
| Age | -0.32619733 | -0.18065171 | -2.9577483 | 1.6000093 | 0 |
| SVS | 3.78903494 | 3.83612295 | 0.9745805 | 7.002577 | 0.81818182 |
| Lev WD | -0.23388257 | -0.15999964 | -1.8319343 | 1.3446853 | 0 |
| Deg WD | -0.26921502 | -0.26994804 | -1.819198 | 1.3103274 | 0 |
| MMSE | 2.67710635 | 2.62961731 | -0.0052082 | 5.7622507 | 0.51515152 |
| AIWD | 1.00825703 | 1.14575585 | 0.0478611 | 1.9940177 | 0 |
| NIHSS | 17.8637582 | 18.1853138 | 12.2800749 | 20.8411582 | 1 |

**Table S6 Variance inflation factor (VIF) values for variables included in the multivariate logistic regression model.**

|  | **VIF** |
| --- | --- |
| NIHSS | 1.083227013 |
| PV | 1.061339392 |
| Sev WD | 1.105445919 |
| MO | 1.062141678 |
| SV 1000 | 1.079403185 |
| AF | 1.067886569 |
| DM | 1.10878013 |
| CSO | 1.095899381 |
| HLD | 1.060396054 |
| HTN | 1.098493242 |

**Table S7 Hyperparameters optimized through Random Search for each ML model.**

| **Model** | **Tuning Method** | **Best Hyperparameters** |
| --- | --- | --- |
| RandomForest | Random Search | {'n_estimators': 100, 'min_samples_split': 10, 'min_samples_leaf': 1, 'max_features': 'sqrt', 'max_depth': 10} |
| XGBoost | Random Search | {'subsample': 0.8, 'n_estimators': 300, 'min_child_weight': 5, 'max_depth': 7, 'learning_rate': 0.1, 'gamma': 0.1, 'colsample_bytree': 1.0} |
| LightGBM | Random Search | {'subsample': 0.8, 'num_leaves': 150, 'n_estimators': 200, 'min_child_samples': 10, 'max_depth': 7, 'learning_rate': 0.01, 'colsample_bytree': 0.6} |
| GradientBoosting | Random Search | {'subsample': 0.8, 'n_estimators': 100, 'min_samples_split': 10, 'min_samples_leaf': 4, 'max_depth': 3, 'learning_rate': 0.01} |
| AdaBoost | Random Search | {'n_estimators': 200, 'learning_rate': 0.1} |
| LogisticReg LinearReg | Random Search | {'C': 1} |
| Lasso | Random Search | {'C': 10} |

**Table S8 Brier scores of eight ML models in the training cohort.**

| Name | bs score |  |  |
| --- | --- | --- | --- |
| RandomForest | brier_score=0.0747(0.0619-0.0875) | | |
| XGBoos | brier_score=0.0508(0.0341-0.0692) | | |
| LightGBM | brier_score=0.0646(0.0526-0.0773) | | |
| GradientBoosting | brier_score=0.1128(0.1011-0.1246) | | |
| AdaBoost | brier_score=0.1470(0.1366-0.1570) | | |
| LogisticReg LinearReg | brier_score=0.1238(0.0988-0.1495) | | |
| Lasso | brier_score=0.1221(0.0946-0.1499) | | |
| GaussianNB | brier_score=0.1491(0.1258-0.1730) | | |

**Table S9 Brier scores of eight ML models in the validation cohort.**

| Name | bs_score |  |  |
| --- | --- | --- | --- |
| RandomForest | brier_score=0.1526(0.1194-0.1886) | | |
| XGBoos | brier_score=0.1465(0.0938-0.1991) | | |
| LightGBM | brier_score=0.1442(0.1086-0.1799) | | |
| GradientBoosting | brier_score=0.1738(0.1471-0.2008) | | |
| AdaBoost | brier_score=0.1766(0.1580-0.1954) | | |
| LogisticReg LinearReg | brier_score=0.1553(0.1106-0.2053) | | |
| Lasso | brier_score=0.1578(0.1098-0.2120) | | |
| GaussianNB | brier_score=0.1684(0.1320-0.2075) | | |

**Table S10 Precision–recall (PR) parameters of eight ML models in the validation cohort.**

| Type | PRAUC | Dataset |
| --- | --- | --- |
| RandomForest | 0.851 | vad |
| XGBoos | 0.884 | vad |
| LightGBM | 0.893 | vad |
| GradientBoosting | 0.827 | vad |
| AdaBoost | 0.881 | vad |
| LogisticReg LinearReg | 0.841 | vad |
| Lasso | 0.84 | vad |
| GaussianNB | 0.845 | vad |

**Table S11 Area under the ROC curve (AUC) with 95% confidence intervals for eight ML models in the training and validation cohorts.**

| **Model** | **dfclass** | **AUC** | **Low** | **Up** |
| --- | --- | --- | --- | --- |
| RandomForest | dev | 0.986 | 0.974 | 0.996 |
| XGBoos | dev | 0.983 | 0.967 | 0.995 |
| LightGBM | dev | 0.99 | 0.979 | 0.997 |
| GradientBoosting | dev | 0.979 | 0.961 | 0.993 |
| AdaBoost | dev | 0.966 | 0.943 | 0.985 |
| LogisticReg LinearReg | dev | 0.904 | 0.86 | 0.944 |
| Lasso | dev | 0.906 | 0.862 | 0.946 |
| GaussianNB | dev | 0.869 | 0.817 | 0.917 |
| RandomForest | vad | 0.865 | 0.774 | 0.942 |
| XGBoos | vad | 0.876 | 0.796 | 0.947 |
| LightGBM | vad | 0.878 | 0.802 | 0.943 |
| GradientBoosting | vad | 0.837 | 0.747 | 0.919 |
| AdaBoost | vad | 0.88 | 0.802 | 0.95 |
| LogisticReg LinearReg | vad | 0.844 | 0.75 | 0.926 |
| Lasso | vad | 0.84 | 0.742 | 0.924 |
| GaussianNB | vad | 0.826 | 0.729 | 0.909 |

**Table S12 Five-fold stratified cross-validation results on the training cohort (n = 199). AdaBoost achieved the highest mean cross-validated AUC (0.930 ± 0.046).**

| **Model** | **Mean AUC** | **SD AUC** | **Mean Sens** | **Mean Spec** | **Mean F1** | **Mean Acc** |
| --- | --- | --- | --- | --- | --- | --- |
| AdaBoost | 0.9299 | 0.0464 | 0.9123 | 0.8235 | 0.8912 | 0.8742 |
| RandomForest | 0.9051 | 0.0358 | 0.8862 | 0.7647 | 0.8579 | 0.8344 |
| GradientBoosting | 0.9135 | 0.0576 | 0.9209 | 0.7059 | 0.8601 | 0.8291 |
| LogisticReg | 0.8675 | 0.0315 | 0.8158 | 0.7176 | 0.8043 | 0.774 |
| Lasso | 0.8649 | 0.0326 | 0.8071 | 0.7176 | 0.7995 | 0.769 |
| GaussianNB | 0.8762 | 0.0281 | 0.8245 | 0.7059 | 0.8064 | 0.7738 |

**Table S13 Bootstrap-derived 95% confidence intervals for all performance metrics in the validation cohort (n = 86; 1,000 bootstrap resamples).**

| **Model** | **AUC-mean** | **AUC-95CI** | **Sens-mean** | **Sens-95CI** | **Spec-mean** | **Spec-95CI** | **F1-mean** | **F1-95CI** | **PPV-mean** | **PPV-95CI** | **NPV-mean** | **NPV-95CI** | **Acc-mean** | **Acc-95CI** | **Brier-mean** | **Brier-95CI** |
| --- | --- | --- | --- | --- | --- | --- | --- | --- | --- | --- | --- | --- | --- | --- | --- | --- |
| AdaBoost | 0.9253 | [0.868-0.971] | 0.9383 | [0.864-1.000] | 0.6406 | [0.500-0.793] | 0.8383 | [0.760-0.907] | 0.7598 | [0.645-0.869] | 0.8955 | [0.765-1.000] | 0.8035 | [0.721-0.884] | 0.1692 | [0.151-0.188] |
| RandomForest | 0.9338 | [0.881-0.979] | 0.9174 | [0.830-0.981] | 0.7703 | [0.634-0.903] | 0.8697 | [0.800-0.934] | 0.8289 | [0.722-0.927] | 0.8847 | [0.765-0.974] | 0.8507 | [0.779-0.919] | 0.1259 | [0.098-0.158] |
| GradientBoosting | 0.9115 | [0.849-0.966] | 0.9376 | [0.860-1.000] | 0.5903 | [0.441-0.744] | 0.8226 | [0.741-0.895] | 0.7349 | [0.621-0.844] | 0.8863 | [0.759-1.000] | 0.7803 | [0.698-0.872] | 0.1617 | [0.135-0.189] |
| LogisticReg | 0.9141 | [0.849-0.969] | 0.9172 | [0.833-0.980] | 0.7728 | [0.641-0.903] | 0.8703 | [0.796-0.932] | 0.8301 | [0.725-0.930] | 0.8851 | [0.771-0.971] | 0.8517 | [0.779-0.919] | 0.1174 | [0.080-0.157] |
| Lasso | 0.9141 | [0.848-0.969] | 0.9172 | [0.833-0.980] | 0.7728 | [0.641-0.903] | 0.8703 | [0.796-0.932] | 0.8301 | [0.725-0.930] | 0.8851 | [0.771-0.971] | 0.8517 | [0.779-0.919] | 0.1155 | [0.076-0.157] |
| GaussianNB | 0.9071 | [0.839-0.964] | 0.8963 | [0.804-0.974] | 0.7728 | [0.641-0.903] | 0.8589 | [0.784-0.926] | 0.8269 | [0.722-0.927] | 0.8602 | [0.742-0.958] | 0.8403 | [0.767-0.907] | 0.1188 | [0.077-0.164] |

**Table S14 Youden-Index Optimized Classification Thresholds Compared with Default 0.5 Threshold (Validation Cohort, n = 86).**

| **Model** | **Threshold** | **Youden J** | **Sens Opt** | **Spec Opt** | **Sens 0.5** | **Spec 0.5** |
| --- | --- | --- | --- | --- | --- | --- |
| AdaBoost | 0.531 | 0.6608 | 0.7959 | 0.8649 | 0.8367 | 0.7838 |
| RandomForest | 0.561 | 0.6944 | 0.7755 | 0.9189 | 0.7959 | 0.8108 |
| GradientBoosting | 0.558 | 0.5256 | 0.7959 | 0.7297 | 0.8367 | 0.6486 |
| LogisticReg | 0.578 | 0.62 | 0.7551 | 0.8649 | 0.7551 | 0.7838 |
| Lasso | 0.434 | 0.5935 | 0.8367 | 0.7568 | 0.7755 | 0.7838 |
| GaussianNB | 0.398 | 0.7496 | 0.9388 | 0.8108 | 0.8367 | 0.8108 |

**Table S15 Inverse-Probability-of-Split-Weighted (IPW) Sensitivity Analysis for AdaBoost Model.**

| **Analysis** | **AUC mean** | **95% CI Lower** | **95% CI Upper** |
| --- | --- | --- | --- |
| Unweighted | 0.8972 | 0.8248 | 0.9572 |
| IPW-Weighted | 0.8971 | 0.8148 | 0.9579 |

**Table S 16 Training–Validation Cohort Split Balance Assessment.**

| **Variable** | **Train** | **Validation** | **Train Mean** | **Val Mean** | **p-value** |
| --- | --- | --- | --- | --- | --- |
| age | 62 | 67 | 68.6 | 69.5 | 0.764 |
| MCA | 0.462 | 0.430 | 0.462 | 0.430 | 0.7115 |
| MO | 0.558 | 0.500 | 0.558 | 0.500 | 0.4418 |
| sex | 0.548 | 0.558 | 0.548 | 0.558 | 0.9742 |
| HTN | 0.523 | 0.500 | 0.523 | 0.500 | 0.8247 |
| DM | 0.492 | 0.442 | 0.492 | 0.442 | 0.5119 |
| AF | 0.518 | 0.558 | 0.518 | 0.558 | 0.6168 |

**Table S17 DeLong Pairwise AUC Comparison with Bonferroni Correction (Validation Cohort, n = 86).**

| **Model 1** | **Model 2** | **AUC 1** | **AUC 2** | **Delta AUC** | **z** | **p raw** | **p Bonferroni** |
| --- | --- | --- | --- | --- | --- | --- | --- |
| AdaBoost | RandomForest | 0.8963 | 0.8809 | 0.0156 | 0.821 | 0.4115 | 1 |
| AdaBoost | GradientBoosting | 0.8963 | 0.8387 | 0.0575 | 2.433 | 0.015 | 0.2245 |
| AdaBoost | LogisticReg | 0.8963 | 0.8632 | 0.0334 | 1.104 | 0.2694 | 1 |
| AdaBoost | Lasso | 0.8963 | 0.8605 | 0.0372 | 1.233 | 0.2175 | 1 |
| AdaBoost | GaussianNB | 0.8963 | 0.904 | -0.0068 | -0.225 | 0.8219 | 1 |
| RandomForest | GradientBoosting | 0.8809 | 0.8387 | 0.0433 | 1.324 | 0.1855 | 1 |
| RandomForest | LogisticReg | 0.8809 | 0.8632 | 0.0184 | 0.802 | 0.4225 | 1 |
| RandomForest | Lasso | 0.8809 | 0.8605 | 0.0204 | 0.863 | 0.3883 | 1 |
| RandomForest | GaussianNB | 0.8809 | 0.904 | -0.0243 | -1.029 | 0.3035 | 1 |
| GradientBoosting | LogisticReg | 0.8387 | 0.8632 | -0.0252 | -0.584 | 0.5591 | 1 |
| GradientBoosting | Lasso | 0.8387 | 0.8605 | -0.022 | -0.489 | 0.6246 | 1 |
| GradientBoosting | GaussianNB | 0.8387 | 0.904 | -0.0642 | -1.429 | 0.1531 | 1 |
| LogisticReg | Lasso | 0.8632 | 0.8605 | 0.0031 | 0.657 | 0.5114 | 1 |
| LogisticReg | GaussianNB | 0.8632 | 0.904 | -0.0407 | -2.465 | 0.0137 | 0.2055 |
| Lasso | GaussianNB | 0.8605 | 0.904 | -0.0434 | -2.482 | 0.0131 | 0.1958 |

**Table S18 Post-hoc Sample Size Assessment Following Riley et al. (BMJ, 2019).**

| **Parameter** | **Value** |
| --- | --- |
| Validation sample size (n) | 86 |
| Number of events | 49 |
| Number of non-events | 37 |
| Events per variable (EPV) | 4.9 |
| AdaBoost validation AUC | 0.8963 |
| Standard error of AUC | 0.0501 |
| Min detectable Delta-AUC (80% power) | 0.1404 |
| Min N for Delta-AUC=0.05 | 292 |

**Table S19 Hosmer–Lemeshow Goodness-of-Fit Test Results for All Models in Both Training and Validation Cohorts.**

| **Model** | **HL Train** | **p Train** | **HL Validation** | **p Validation** |
| --- | --- | --- | --- | --- |
| AdaBoost | 69.013 | 0 | 17.15 | 0.0007 |
| RandomForest | 34.575 | 0 | 5.946 | 0.1143 |
| GradientBoosting | 61.61 | 0 | 11.717 | 0.0084 |
| LogisticReg | 9.586 | 0.2953 | 1.142 | 0.767 |
| Lasso | 8.119 | 0.4219 | 2.599 | 0.4577 |
| GaussianNB | 2.658 | 0.9539 | 2.979 | 0.3949 |

**Table S20 Complete confusion matrix-derived metrics for all models in both training and validation cohorts.**

| **Model** | **Cohort** | **TP** | **TN** | **FP** | **FN** | **Sens** | **Spec** | **PPV** | **NPV** | **Acc** | **F1** | **AUC** |
| --- | --- | --- | --- | --- | --- | --- | --- | --- | --- | --- | --- | --- |
| AdaBoost | Training | 104 | 74 | 11 | 10 | 0.9123 | 0.8706 | 0.9043 | 0.881 | 0.8945 | 0.9083 | 0.9634 |
| AdaBoost | Validation | 44 | 25 | 14 | 3 | 0.9362 | 0.641 | 0.7586 | 0.8929 | 0.8023 | 0.8381 | 0.9244 |
| RandomForest | Training | 108 | 75 | 10 | 6 | 0.9474 | 0.8824 | 0.9153 | 0.9259 | 0.9196 | 0.931 | 0.9792 |
| RandomForest | Validation | 43 | 30 | 9 | 4 | 0.9149 | 0.7692 | 0.8269 | 0.8824 | 0.8488 | 0.8687 | 0.9326 |
| GradientBoosting | Training | 110 | 70 | 15 | 4 | 0.9649 | 0.8235 | 0.88 | 0.9459 | 0.9045 | 0.9205 | 0.9786 |
| GradientBoosting | Validation | 44 | 23 | 16 | 3 | 0.9362 | 0.5897 | 0.7333 | 0.8846 | 0.7791 | 0.8224 | 0.9108 |
| LogisticReg | Training | 97 | 67 | 18 | 17 | 0.8509 | 0.7882 | 0.8435 | 0.7976 | 0.8241 | 0.8472 | 0.8917 |
| LogisticReg | Validation | 43 | 30 | 9 | 4 | 0.9149 | 0.7692 | 0.8269 | 0.8824 | 0.8488 | 0.8687 | 0.9124 |
| Lasso | Training | 96 | 67 | 18 | 18 | 0.8421 | 0.7882 | 0.8421 | 0.7882 | 0.8191 | 0.8421 | 0.8918 |
| Lasso | Validation | 43 | 30 | 9 | 4 | 0.9149 | 0.7692 | 0.8269 | 0.8824 | 0.8488 | 0.8687 | 0.9124 |
| GaussianNB | Training | 96 | 67 | 18 | 18 | 0.8421 | 0.7882 | 0.8421 | 0.7882 | 0.8191 | 0.8421 | 0.8913 |
| GaussianNB | Validation | 42 | 30 | 9 | 5 | 0.8936 | 0.7692 | 0.8235 | 0.8571 | 0.8372 | 0.8571 | 0.9053 |

**Table S21 Optimal decision thresholds determined by Youden Index maximization for all models in the validation cohort.**

| **Model** | **Optimal Threshold** | **Youden** | **Sens Opt** | **Spec Opt** |
| --- | --- | --- | --- | --- |
| AdaBoost | 0.525 | 0.7054 | 0.9362 | 0.7692 |
| RandomForest | 0.586 | 0.7185 | 0.8723 | 0.8462 |
| GradientBoosting | 0.677 | 0.689 | 0.766 | 0.9231 |
| LogisticReg | 0.627 | 0.7229 | 0.8511 | 0.8718 |
| Lasso | 0.657 | 0.7229 | 0.8511 | 0.8718 |
| GaussianNB | 0.637 | 0.7185 | 0.8723 | 0.8462 |

**Table S22 Subgroup analysis of AdaBoost model performance stratified by key clinical and imaging variables in the validation cohort.**

| **Subgroup** | **N** | **Events** | **AUC** | **Sensitivity** | **Specificity** | **F1** |
| --- | --- | --- | --- | --- | --- | --- |
| Sex=No | 35 | 19 | 0.967 | 0.947 | 0.75 | 0.878 |
| Sex=Yes | 51 | 28 | 0.883 | 0.929 | 0.565 | 0.812 |
| HTN=No | 38 | 13 | 0.954 | 1.0 | 0.64 | 0.743 |
| HTN=Yes | 48 | 34 | 0.892 | 0.912 | 0.643 | 0.886 |
| AF=No | 40 | 14 | 0.893 | 0.929 | 0.615 | 0.703 |
| AF=Yes | 46 | 33 | 0.959 | 0.939 | 0.692 | 0.912 |
| DM=No | 52 | 29 | 0.908 | 0.897 | 0.652 | 0.825 |
| DM=Yes | 34 | 18 | 0.983 | 1.0 | 0.625 | 0.857 |
| MO=No | 48 | 14 | 0.89 | 0.857 | 0.676 | 0.649 |
| MO=Yes | 38 | 33 | 0.848 | 0.97 | 0.4 | 0.941 |
| Sev WD=No | 50 | 21 | 0.901 | 0.857 | 0.69 | 0.75 |
| Sev WD=Yes | 36 | 26 | 0.919 | 1.0 | 0.5 | 0.912 |

**Table S23 The table of the top ten strongest feature interactions.**

|  | **Strength** |
| --- | --- |
| HTN → MO | 0.1030 |
| HLD → Sev WD | 0.0820 |
| Sev WD → NIHSS | 0.0687 |
| AF → NIHSS | 0.0675 |
| AF → MO | 0.0499 |
| PV → SV 1000 | 0.0429 |
| HLD → AF | 0.0323 |
| PV → Sev WD | 0.0274 |
| PV → MO | 0.0250 |
| MO → NIHSS | 0.0228 |
